# Examining the Reticulocyte Preference of Two *Plasmodium berghei* Strains during Blood-Stage Malaria Infection

**DOI:** 10.3389/fmicb.2018.00166

**Published:** 2018-02-20

**Authors:** Neha Thakre, Priyanka Fernandes, Ann-Kristin Mueller, Frederik Graw

**Affiliations:** ^1^Centre for Modeling and Simulation in the Biosciences, BioQuant-Center, Heidelberg University, Heidelberg, Germany; ^2^Parasitology Unit, Centre for Infectious Diseases, University Hospital, Heidelberg, Germany; ^3^German Center for Infectious Diseases (DZIF), Heidelberg, Germany

**Keywords:** Malaria, *Plasmodium*, mathematical modeling, infection dynamics, parasite infectivity, erythropoiesis

## Abstract

The blood-stage of the *Plasmodium* parasite is one of the key phases within its life cycle that influences disease progression during a malaria infection. The efficiency of the parasite in infecting red blood cells (RBC) determines parasite load and parasite-induced hemolysis that is responsible for the development of anemia and potentially drives severe disease progression. However, the molecular factors defining the infectivity of *Plasmodium* parasites have not been completely identified so far. Using the *Plasmodium berghei* mouse model for malaria, we characterized and compared the blood-stage infection dynamics of *Pb*ANKA WT and a mutant parasite strain lacking a novel *Plasmodium* antigen, *Pb*maLS_05, that is well conserved in both human and animal *Plasmodium* parasite strains. Infection of mice with parasites lacking *PbmaLS_05* leads to lower parasitemia levels and less severe disease progression in contrast to mice infected with the wildtype *Pb*ANKA strain. To specifically determine the effect of deleting *PbmaLS_05* on parasite infectivity we developed a mathematical model describing erythropoiesis and malarial infection of RBC. By applying our model to experimental data studying infection dynamics under normal and drug-induced altered erythropoietic conditions, we found that both *Pb*ANKA and *PbmaLS_05* (-) parasite strains differed in their infectivity potential during the early intra-erythrocytic stage of infection. Parasites lacking *PbmaLS_05* showed a decreased ability to infect RBC, and immature reticulocytes in particular that are usually a preferential target of the parasite. These altered infectivity characteristics limit parasite burden and affect disease progression. Our integrative analysis combining mathematical models and experimental data suggests that deletion of *PbmaLS_05* affects productive infection of reticulocytes, which makes this antigen a useful target to analyze the actual processes relating RBC preferences to the development of severe disease outcomes in malaria.

## Introduction

Malaria caused by the *Plasmodium* parasite is one of the most serious tropical diseases with a major impact on global health. In 2015, malaria was responsible for 212 million clinical cases and an estimated number of 429,000 deaths worldwide (World Health Organization, 2016).

Within the host, *Plasmodium* parasites follow a complex life cycle involving parasite replication and differentiation in liver and blood (Portugal et al., 2011). Disease progression is mainly associated with the blood-stage of the parasite, as parasite-induced infection and lysis of red blood cells (RBC) leads to the development of anemia (Dondorp et al., 1999), one of the main symptoms characterizing a malaria infection.

Many *Plasmodium* parasite strains have been found to differ in their infectivity during the blood-stage infection phase by targeting RBC of different ages (McQueen and McKenzie, 2004). Several parasite species express a preference for immature RBC (reticulocytes) compared to mature RBC (erythrocytes/normocytes). Estimates indicate a 34- to 180-fold higher preference in *Plasmodium vivax* (Mons et al., 1988; Mons, 1990) and a 1.6- to 14-fold preference in *Plasmodium falciparum* in humans (Wilson et al., 1977; Pasvol et al., 1980; Clough et al., 1998), with the latter one being responsible for cerebral malaria, a severe neuropathy resulting in death or severe neurological sequelae in survivors (Seydel et al., 2015; Gupta et al., 2017). In rodents, strains of *Plasmodium chabaudi* show such age-specific targeting of RBC during the acute infection phase (Antia et al., 2008), while *Plasmodium berghei* (Singer et al., 1955; McNally et al., 1992; Sexton et al., 2004; Cromer et al., 2006, 2009) has an estimated ~150-fold preference for reticulocytes during the late stages of infection (Cromer et al., 2006). It has been suggested that high reticulocyte preference is responsible for the highest parasite densities which in turn induce severe anemia (McQueen and McKenzie, 2004), i.e., with anemia-induced production of novel reticulocytes conversely fueling parasite replication. However, which factors govern and influence the infectivity of parasites and to which extent elevated parasite densities might also influence faster disease progression have not been determined so far (Beeson et al., 2016).

In this context, *Pb*maLS_05 was identified as a novel *Plasmodium* antigen that plays an important role in the development of experimental cerebral malaria (ECM) (Fernandes et al., submitted manuscript), a neuropathology that is characteristically similar to human cerebral malaria (de Souza et al., 2010; Hoffmann et al., 2016). The gene is well conserved in human and rodent *Plasmodium* strains and as it is expressed during both late intra-hepatic and intra-erythrocytic stages of the parasite, this cross-stage antigen represents a potential vaccine target. The protein localizes to the apicoplast organelle—an endosymbiotic relict of the parasite that is important for intra-erythrocytic survival. Deletion of *PbmaLS_05* was suggested to influence parasite replication or viability in the blood (Fernandes et al., submitted manuscript), but the effects on infectivity and potential cell preferences are not known.

Determining a parasite's infectivity potential during the intra-erythrocytic stage requires the disentangling of parasite replication dynamics and infection-induced changes to erythropoiesis. Mathematical modeling has been an essential tool to analyze these processes. In addition to detecting target cell preferences and differences in infection profiles of various pathogens, mathematical models allow us to specifically account for the processes of erythropoiesis, parasite infection and turnover, as well as disease-induced anemia (McQueen and McKenzie, 2004; Cromer et al., 2006, 2009; Antia et al., 2008; Fonseca and Voit, 2015). There have been various modeling approaches describing the blood-stage infection dynamics of different *Plasmodium* parasite strains in various levels of detail (Antia et al., 2008; Mideo et al., 2008; Cromer et al., 2009; Li et al., 2011).

In this study, we used a combination of different experimental protocols and mathematical models to investigate parasite blood-stage infection dynamics under physiological and drug-induced altered erythropoietic conditions to elucidate the effects of deletion of *PbmaLS_05* (KO) on parasite infectivity. We concentrated on the acute phase of infection, analyzing the first 4 days after infection with parasitized RBC until the time when mice infected by the *Pb*ANKA (WT) strain showed first signs of ECM. Our age-structured model explicitly accounts for RBC development and erythropoiesis and is thereby able to determine possible target cell preferences for both parasite strains. Our results indicate dynamic malaria-induced changes to erythropoiesis during disease progression and suggest that deletion of *Pb*maLS_05 has an effect on the productive infection of reticulocytes.

## Materials and methods

### Ethics statement

All animal experiments were performed according to European regulations concerning FELASA category B and GV-SOLAS standard guidelines. Animal experiments were approved by German authorities (Regierungspräsidium Karlsruhe, Germany), § 8 Abs. 1 Tierschutzgesetz (TierSchG) under the license G-260/12 and were performed according to National and European regulations. For all experiments, female C57BL/6 mice (6- to 8-week-old) were purchased from Janvier laboratories, France. All mice were kept under specified pathogen-free (SPF) conditions within the animal facility at Heidelberg University (IBF).

### Experimental protocol and data

**Figure 1:**
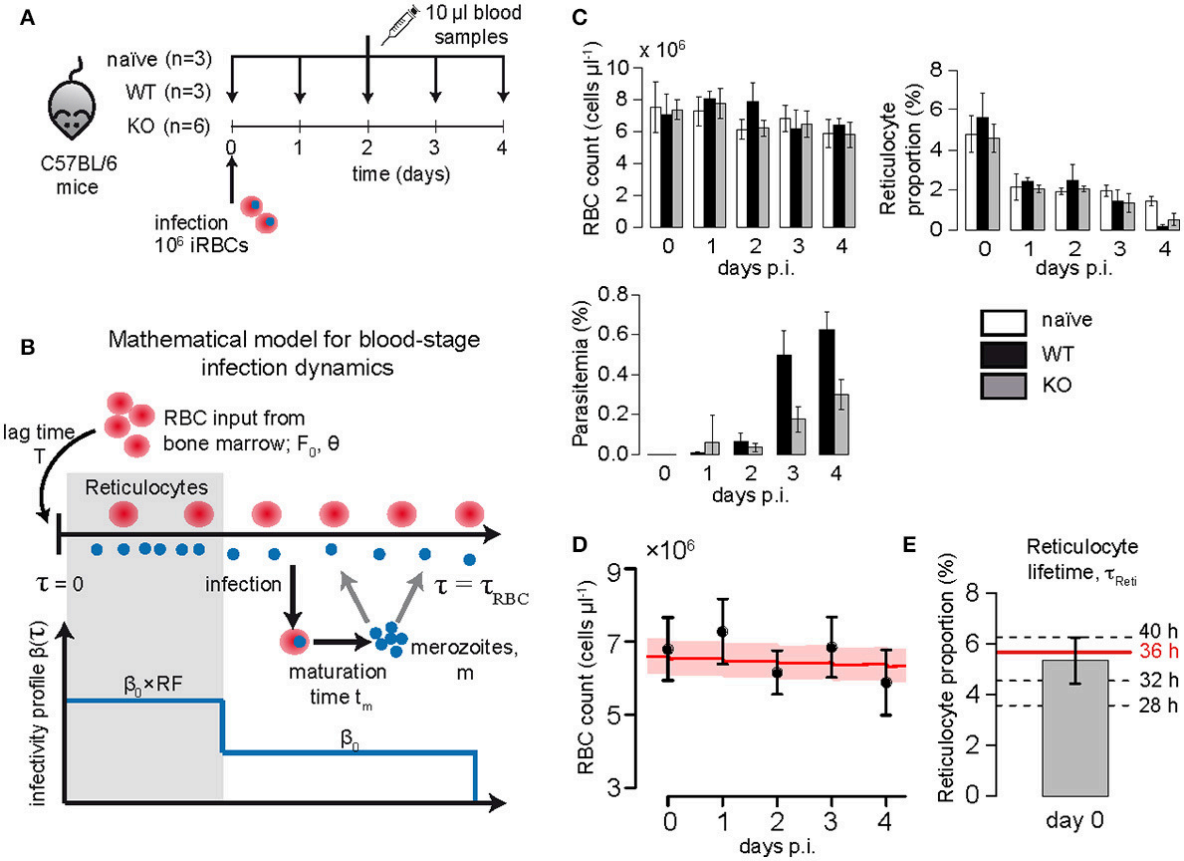
**(A)** Experimental protocol: C57BL/6 mice were infected with 10^6^ iRBC of *Pb*ANKA (WT), *PbmaLS_05* (-) (KO) or left uninfected. Daily samples of 10 μl blood were drawn to measure the concentration of RBC (cells/μl), reticulocyte proportion and parasitemia (in % of RBC). **(B)** Sketch of the mathematical model describing erythropoiesis and blood-stage infection dynamics of the parasite. For a detailed description of the model see section Materials and Methods. **(C)** Measured concentration of RBC (cells/μl), reticulocyte proportion (in % of RBC) and parasitemia (in % of RBC) for each of the different groups analyzed. **(D)** The plot shows the measured concentration of red blood cells for naïve mice (mean + SD, *n* = 3), as well as the dynamics predicted by our model (best fit-red solid line, 95%-confidence interval- shaded area) using parameter estimates for RBC turnover and reticulocyte production as given in Table 1. **(E)** Based on model predictions and the measured proportion of reticulocytes on day 0, we consider a maturation time for reticulocytes of τ_*Reti*_ = 36 h.

In the first set of experiments, C57BL/6 mice were intravenously infected with 10^6^ infected red blood cells (iRBC) taken from mice infected either with wild-type *Pb*GFP Luc_con_ (*P. berghei* line 676m1c11) (WT), a GFP-luciferase transgenic derivative of *P. berghei* ANKA (Franke-Fayard et al., 2005), or the mutant *PbmaLS_05* (–) parasites (KO) generated in the wild-type *Pb*GFP Luc_con_ strain (Fernandes et al., submitted manuscript). An additional group of age-matched mice was left uninfected and treated as naïve controls. Daily blood samples of 10 μl were taken from all mice from the day of infection until day 4 post infection (p.i.). The total red blood cell count and reticulocyte percentage were measured using a Coulter counter and FACS analysis of CD71 (CD71-PE, eBioscience, Clone R17217) labeled reticulocytes, respectively. Parasitemia was determined by FACS analysis of GFP positive infected red blood cells. A sketch of the experimental protocol is shown in Figure 1A. Mice were sacrificed at day 5 p.i., when mice infected with WT parasites showed first symptoms of ECM.

A second set of mice were pretreated with two doses of phenylhydrazine (PHZ, 40 mg/kg), on two consecutive days prior to infection with 10^6^ iRBC using the same groups of mice as before. Again, daily blood samples of 10 μl were taken from each mouse and analyzed up to day 5 p.i. before sacrificing the mice on day 6 p.i.

### Mathematical model for erythropoiesis and blood-stage infection dynamics

To describe the blood stage-infection dynamics of the murine malaria parasite accounting for RBC age, we used a mathematical model for erythropoiesis as described before (Mackey, 1997). The age-structured model follows the population density of RBCs of age τ at time *t* based on a system of coupled ordinary differential equations that breaks the age ranges of RBC into *n* = τ_*RBC*_/*h* different compartments with *h* being the compartment size and τ_*RBC*_ the maximal lifespan of RBCs. The concentration of RBCs within each compartment is denoted by *x*_*i*_*(t), i* = *1,… n*. New RBCs are constantly produced by the bone marrow that enter the population of RBCs after a delay *T*, with the actual influx at each time point determined by a Hill-function dependent on the maximal production rate of RBCs in the bone marrow, *F*_0_, and the concentration of RBCs at time *t-T, X(t-T)*. Mathematically, the model is then described by the following system of ordinary differential equations:

(1) $$\begin{array}{l} \frac{dx_{1}}{dt}=F_{0}\frac{\theta^{k}}{\theta^{k}+{\left(X\left(t-T\right)\right)}^{k}}-\;\frac{1}{h}x_{1}\left(t\right)-\frac{1}{\tau_{RBC}}x_{1}\left(t\right) \end{array}$$

(2) $$\begin{array}{l} \frac{dx_{i}}{dt}=\frac{1}{h}\left(x_{i-1}\left(t\right)-x_{i}\left(t\right)\right)-\frac{1}{\tau_{RBC}}x_{i}\left(t\right),\;i=2,\;\ldots ,n \end{array}$$

(3) $$\begin{array}{l} X\left(t\right)=\sum_{i=1}^{n}x_{i}(t) \end{array}$$

Hereby, the parameter θ describes the concentration of RBC where the production rate is half of the maximum and *k* the Hill-coefficient (Mackey, 1997). In addition, we also assumed that in each compartment *x*_*i*_ RBCs are lost by an age-independent loss-rate 1/τ_*RBC*_ to have at least 85% of RBC lost until their assumed maximal lifespan τ_*RBC*_. Equations (1–3) represent a mean-field approximation of the originally developed system relying on partial differential equations, thereby transforming assumed fixed, constant lifespans of RBC into gamma-distributed lifetimes (Mackey, 1997; Antia et al., 2008).

This basic model for erythropoiesis is then extended to account for malaria blood-stage infection as done previously (McQueen and McKenzie, 2004; Antia et al., 2008; Figure 1B). Uninfected RBCs can get infected by free merozoites, *z*, at a rate β(τ), which is dependent on the age-preference of the infecting parasite strain. Each infected RBC releases a number of merozoites, *m*, by bursting after having reached an infection maturation time, *t*_*m*_. In addition, free merozoites are assumed to have an average lifetime of 1/*d*_*m*_. As for uninfected RBC, the concentration of infected cells, *Y(t)*, is broken down into *g* = *t*_*m*_*/h* different age compartments, *y*_*i*_*(t), i* = *1,…,g* leading to a system of coupled ordinary differential equations with a gamma-distributed maturation time with mean *t*_*m*_. The basic model for erythropoiesis (Equations 1–3) is then extended to:

(4) $$\begin{array}{l} \frac{dx_{1}}{dt}=F_{0}\frac{\theta^{k}}{\theta^{k}+{\left(X\left(t-T\right)\right)}^{k}}\;-\;\frac{1}{h}x_{1}\left(t\right)\\ \;-\frac{1}{\tau_{RBC}}x_{1}\left(t\right)-\;\beta_{1}z\left(t\right)x_{1}\left(t\right) \end{array}$$

(5) $$\begin{array}{l} \frac{dx_{i}}{dt}=\frac{1}{h}\left(x_{i-1}\left(t\right)-x_{i}\left(t\right)\right)-\frac{1}{\tau_{RBC}}x_{i}\left(t\right)\\ \;-\;\beta_{i}z\left(t\right)x_{i}\left(t\right),\;i=2,\;\ldots ,n \end{array}$$

(6) $$\begin{array}{l} \frac{dy_{1}}{dt}=\sum_{i=1}^{n}\beta_{i}z(t)x_{i}\left(t\right)-\frac{1}{h}y_{1}\left(t\right) \end{array}$$

(7) $$\begin{array}{l} \frac{dy_{i}}{dt}=\frac{1}{h}\left(y_{i-1}\left(t\right)-y_{i}\left(t\right)\right),\;i=2,\ldots ,\;g \end{array}$$

(8) $$\begin{array}{l} \frac{dz}{dt}=\frac{m}{h}y_{g}\left(t\right)-\sum_{i=1}^{n}\beta_{i}z\left(t\right)x_{i}\left(t\right)-d_{m}z\left(t\right) \end{array}$$

(9) $$\begin{array}{l} \beta_{i}=\{\begin{array}{cc} \beta_{0}RF, & \;i\leq \tau_{Reti}/h\\ \beta_{0}, & i>\tau_{Reti}/h \end{array} \end{array}$$

Hereby, *z(t)* describes the concentration of merozoites at time *t* and RF the so called reticulocyte factor, i.e., the fold-change in infectivity of the parasite for reticulocytes compared to the general infection rate assumed for normocytes, β_0_ (see Cromer et al., 2006). The parameter τ_*Reti*_ defines the maturation time of reticulocytes into normocytes.

### Calculating the average infectivity and reticulocyte preference

In order to compare parasite strains with possible different values for the infection rate, β_0_, and the reticulocyte factor *RF*, we calculated an average infectivity β, which is defined as the infection rate of a single merozoite when placed into the erythropoietic system at the initiation of infection. In a naïve mouse, on average 5.8% of the RBC are reticulocytes, thus the average infectivity is calculated by β = β_0_(0.058*RF* + 0.942).

Besides the reticulocyte factor, *RF*, the reticulocyte preference, *RP*, is calculated based on the ratio between the percentage of infected reticulocytes (relative to all reticulocytes) and the percentage of infected normocytes (relative to all normocytes). Thus, if *R* and *I*_*R*_ define the concentration of reticulocytes and infected reticulocytes, respectively, and *N* and *I*_*N*_ the corresponding concentrations for normocytes, the reticulocyte preference is calculated by *RP* = (*I*_*R*_*/R*)/(*I*_*N*_*/N*). In contrast to the reticulocyte factor, the reticulocyte preference can be directly calculated from experimental measurements.

### Modeling the effect of phenylhydrazine treatment on erythropoiesis

Treatment with Phenylhydrazine (PHZ) is used for experimental induction of anemia in animal models to study hemolytic anemia or anemia caused by destruction or removal of RBCs from the bloodstream (Berger, 2007). Previous studies developed mathematical models to determine and quantify the effect of PHZ on the RBC age distribution and altered erythropoiesis (Savill et al., 2009). However, these models were inadequate to describe our experimental data suggesting that they incompletely addressed the effects of PHZ. To this end, we tested several different known hypotheses for the effect of PHZ on erythropoiesis (Jain and Hochstein, 1980; Berger, 2007; Savill et al., 2009; Moreau et al., 2012) by fitting them to the data of the PHZ-control group (see Supplementary Material Text S3). The models best explaining the experimental data included the following drug effects: (i) Treatment by PHZ leads to instantaneous lyses of a fraction ρ(τ) of RBCs at the time of treatment, *t*_*p*_. Hereby, the effect of lysis depends on the age of the RBC, τ, with normocytes being more strongly affected than reticulocytes (Jain and Hochstein, 1980). (ii) An additional influx of reticulocytes from extra medullary sites is considered at a constant rate *N*_*p*_ with a time-delay *T*_*p*_ after the initiation of treatment to account for stress-induced erythropoiesis. Under severe anemia, such as that induced by PHZ-treatment, extra-medullary sites of erythropoiesis such as the spleen and liver are observed to show an increased contribution of RBCs to circulation (Spivak et al., 1973; Ploemacher et al., 1977; Kim, 2010). Thus, under PHZ-treatment, Equations (1, 2) describing RBC turnover are changed as follows:

(10) $$\begin{array}{l} \frac{dx_{1}}{dt}=F(t)-\left(\frac{1}{h}-\frac{1}{\tau_{RBC}}\right)x_{1}\left(t\right)\\ \;-\rho_{1}I(t=T_{p})\;x_{1}\left(t\right) \end{array}$$

(11) $$\begin{array}{l} \frac{dx_{i}}{dt}=\frac{1}{h}\left(x_{i-1}\left(t\right)-x_{i}\left(t\right)\right)-\frac{1}{\tau_{RBC}}x_{i}\left(t\right)\\ \;-\rho_{i}I(t=T_{p})\;x_{i}\left(t\right),\;i=2,\;\ldots ,n \end{array}$$

(12) $$\begin{array}{l} \rho_{i}=\{\begin{array}{cc} \rho_{0}\gamma , & \;i\;\leq \;\tau_{Reti}/h\\ \rho_{0}, & i\;>\;\tau_{Reti}/h \end{array} \end{array}$$

(13) $$\begin{array}{l} F\left(t\right)=\{\begin{array}{ll} F_{0}\frac{\theta_{k}}{\theta_{k}+{\left(X\left(t-T\right)\right)}^{k}}, & t\;\leq \;t_{p}+T_{p}\\ F_{0}\frac{\theta_{k}}{\theta_{k}+{\left(X\left(t-T\right)\right)}^{k}}+N_{p}, & t>\;t_{p}+T_{p} \end{array} \end{array}$$

Hereby, ρ_0_ defines the fraction of normocytes lysed by PHZ and γ represents the relative comparison of this fraction for reticulocytes. In addition, *I(t* = *T*_*p*_*)* defines the Indicator function, i.e., with *I(t* = *T*_*p*_*)* = 1 if *t* = *T*_*p*_ and 0 otherwise. A sketch of the effects of PHZ treatment on erythropoiesis is shown in **Figure 4A**. A detailed derivation of the model can be found in the Supplementary Material. During infection, we assume that malaria induced changes to RBC production affects both sources of novel reticulocytes, i.e., bone marrow and extra medullary sites alike.

### Model evaluation and fitting procedures

The mathematical models described above were implemented and analyzed using the **R** language of statistical computing (R Development Core Team, 2017). As indicated, the age of uninfected and infected RBC was compartmentalized leading to a tractable system of coupled ordinary differential equations with gamma-distributed lifetimes and maturation times for RBC and infected cells, respectively (Antia et al., 2008). In the following we used a compartment size of 4 h.

The differential equations were solved using the **deSolve** package and models were fitted to the experimental data using the *optim*-fitting routine in **R**. In cases where a strong correlation between parameters hindered convergence of fitting algorithms, a parameter sweep was performed to find combinations of parameters that fit the data. Proportion data (parasitemia levels and proportion of reticulocytes) were *logit*- transformed to allow for normally distributed residuals. Model performance was assessed based on simultaneous fitting for all obtained measurements including RBC concentration, reticulocyte proportion and, where applicable, parasitemia. Blood stage infection dynamics of parasites were determined in a stepwise approach: Parameters describing erythropoiesis were fixed to the indicated values obtained from the naïve control group before analyzing infection dynamics (Table 1). Therefore, measurements for the infection groups, i.e., reticulocyte proportion and RBC count, were scaled relative to the naïve group data when estimating parasite infectivity. To evaluate model performance, the average residual sum of squares (aRSS) was used which is the residual sum of squares divided by the number of data points.

**Table 1:** Estimated parameter values describing erythropoiesis in mice based on the model as described in Equations (1–3) in section Materials and Methods.

**Parameter**	**Description**	**Unit**	**Value**	**References/Comparison**
**ERYTHROPOIESIS**				
*F_0_*	RBC production rate in Bone marrow	(×10^4^) cells μl^−1^ h^−1^	5.95 (4.02, 6.82)	Mackey, 1997
θ	RBC concentration at which half of max. RBC production is reached	(×10^6^) cells μl^−1^	6.65 (5.28, 6.84)	Mackey, 1997
*T*	Delay in RBC production feedback	days	2	Mackey, 1997
*τ_*Reti*_*	Maturation time of reticulocytes in the blood	hours	36	Gronowicz et al., 1984; Wiczling and Krzyzanski, 2008
*τ_*RBC*_*	Lifetime of RBC	days	40	Bannerman, 1983
*k*	Hill-coefficient for RBC feedback		7.6	Mackey, 1997
**DISEASE-INDUCED FEEDBACK MODULATION**				
λ	Loss-rate of gene-expression	day^−1^	2.22 (1.31, 3.05)	
*t_0_*	Time at which half of the max. gene expression is reached	days	3.70 (3.28, 4.23)	
**PARASITE INFECTION**				
*t_*m*_*	Maturation time of iRBC	days	1	Cox, 1988; De Roode, 2004
*m*	Average number of merozoites released per burst		9	Cox, 1988; De Roode, 2004
*d_*m*_*	Clearance rate of merozoites	day^−1^	48	Garnham, 1966

**Figure 2:**
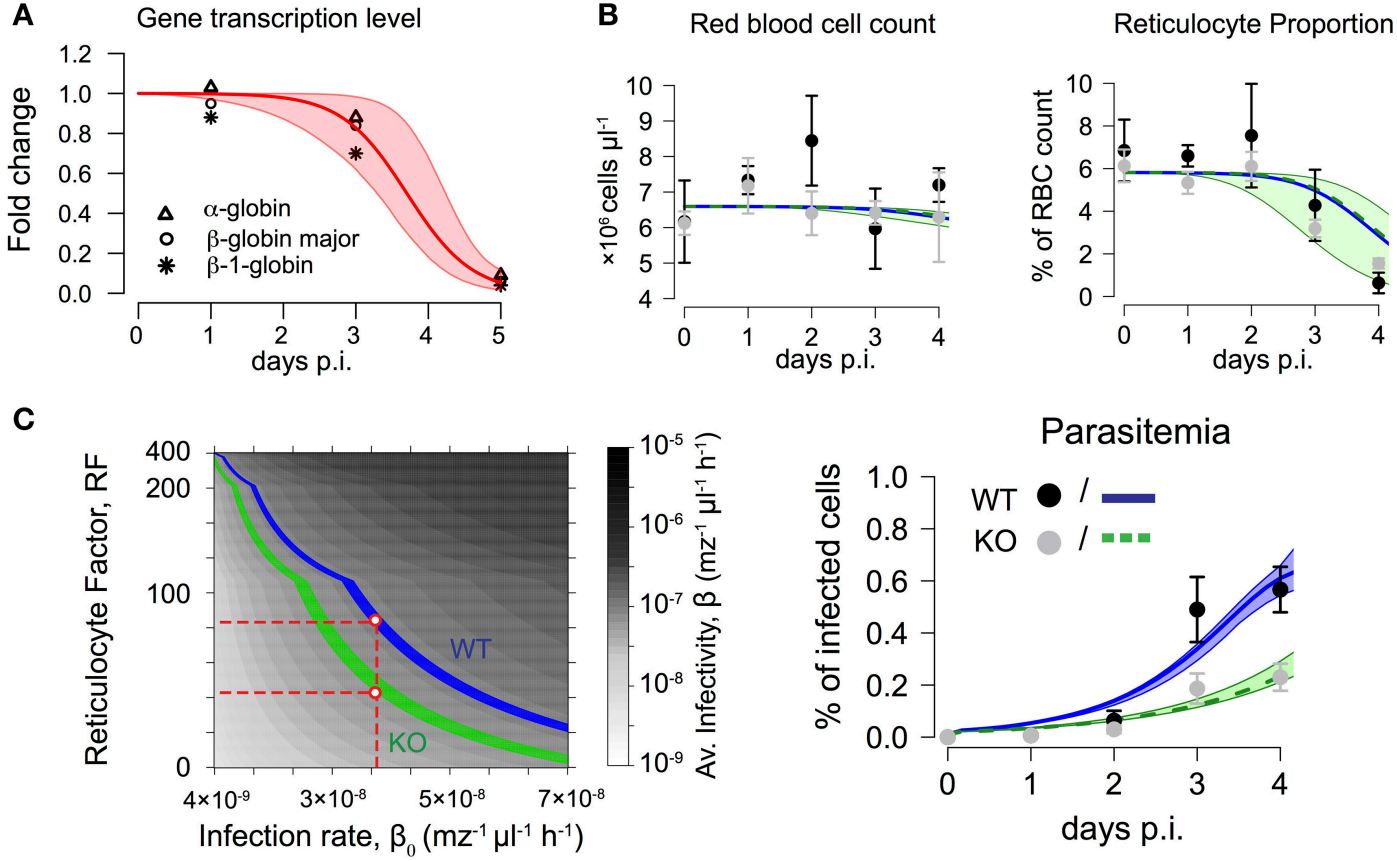
**(A)** Fold change in expression levels of genes associated with erythropoiesis during malaria infection. Symbols represent gene-expression levels of α-globin (Δ), β-globin major (°) and β−1-globin (_*_) as measured in Sexton et al. (2004). Dynamics can be described by a logistic-loss function with *F*(*t*) = (*1-exp*(-λ*t*_0_))/(*1*+*exp*(-λ(*t-t*_0_))) (see main text). Red solid line indicates best fit with λ = 2.22 d^−1^ and *t*_0_ = 3.70 days based on 10^4^ bootstrap replicates simulated from the distribution given by the gene expression levels at each time point (shaded area – 95% confidence interval). **(B)** Dynamics of red blood cell concentration, reticulocyte proportion and parasitemia for mice infected by either WT, *n* = 3 or KO, *n* = 6. The mean and standard deviation for each group (WT- black, KO- gray) are shown. Model results simultaneously predicting the dynamics of all 3 measurements indicate a lower average infectivity for the WT (blue line) compared to KO (green line). Shaded areas indicate 95%-confidence intervals. Corresponding parameter estimates are shown in Table 2. **(C)** Obtained parameter combinations for reticulocyte factor *RF* and infection rate β_0_ indicate a lower average infectivity β per merozoite per hour for the KO parasite compared to the WT. KO parasites have lower reticulocyte factors than the WT if similar infection rates β_0_ for both parasites are assumed (red dashed lines).

*Numbers in brackets represent 95%-confidence intervals of estimates obtained based on the profile likelihood method. In addition, the table contains the parameter estimates for the disease-induced modulation of the RBC feedback dynamics (see Figure 2A), as well as the parameters that were fixed when analyzing the infectivity of the two different parasite strains*.

The 95%-confidence intervals, as well as identifiability of parameter estimates were assessed by profile likelihood analysis (Raue et al., 2009). For the measured data, we report mean and standard error.

## Results

### Characterizing the dynamics of erythropoiesis and determining reticulocyte maturation times in the blood

To determine the dynamics of erythropoiesis in our experimental system, we fitted a mathematical model describing RBC production and subsequent aging (see Equations 1–3 in Materials and Methods; Mary et al., 1980; Mackey, 1997) to the observed progression of RBC concentration in uninfected mice that were sampled daily for 10 μl of blood (see Materials and Methods and Figures 1A–C). In general, bleeding leads to a decrease in the RBC concentration triggering the production of novel RBCs in the bone marrow that will enter the blood circulation after a time delay *T*. Thereby, the magnitude of the feedback depends on the severity of the anemia, i.e., the larger the loss of blood the larger the subsequent RBC production, which is accounted for in our model by a Hill-type function (Mackey, 1997). Assuming a maximal lifespan for RBC of τ_*RBC*_ = 40 days (Bannerman, 1983) and a Hill-coefficient of *k* = 7.6 (Mackey, 1997), we estimated a maximal RBC production rate in the bone marrow of *F*_0_ = 5.95 × 10^4^ cells μl^−1^ h^−1^ [4.02, 6.82] with half of the maximal production rate reached at a RBC concentration of θ = 6.65 × 10^6^ cells μl^−1^[5.28, 6.84], which is approximately 95% of the RBC concentration at steady state. Newly produced red blood cells are estimated to appear in the circulation after a lag-time of *T* = 2 days, testing different possible lag-times including *T* = 0, 1, 2, and 2.5 days. All our estimates are in agreement to parameters that have been determined previously for erythropoiesis in mice (Mary et al., 1980; Mackey, 1997; Figure 1D and Table 1).

As we were especially interested in the dynamics of reticulocytes, i.e., immature red blood cells, we compared model predictions for the proportion of different RBC age classes to the measured proportion of reticulocytes in order to determine the time these cells spend in the blood. We found that a maturation time for reticulocytes into normocytes in the blood of τ_*Reti*_ = 36 h best described our measured proportion of reticulocytes (Figure 1E), which is in agreement to previous calculations determining a maturation time for reticulocytes between 1 and 3 days (Ganzoni et al., 1969; Gronowicz et al., 1984; Wiczling and Krzyzanski, 2008). Thus, for the following analyses we assume that after appearance in the blood, a reticulocyte will take on average 1.5 days to develop into a normocyte.

### Parasite-induced cell death cannot explain the observed loss in reticulocyte proportion

In order to compare the blood-stage infection dynamics of the two *Plasmodium berghei* strains investigated, mice were either infected with *Pb*ANKA (WT) or *PbmaLS*_05 (-) (KO) infected red blood cells and sampled daily for 10 μl of blood. For both strains, we observe a substantial loss in the proportion of reticulocytes around day 3 post infection (p.i.) coinciding with an increase in parasitemia (Figure 1C). At day 4 p.i., when mice infected with WT show first signs of ECM, the parasitemia was approximately twice as high as the one measured for mice infected with the KO (0.63 ±0.05% WT compared to 0.29 ±0.03% KO) (Figure 1C).

To determine systematic differences in the infection dynamics between the two parasite strains, we extended our mathematical model describing erythropoiesis to include malaria blood-stage infection dynamics (see Equations 4–9). Hereby, RBCs get infected by merozoites at an infection rate β and infected RBC (iRBC) will release new merozoites *m* after a certain maturation time *t*_*m*_ (see Figure 1B and Materials and Methods for a detailed explanation of the extended model). Assuming the average lifespan of a merozoite of 1/*d*_*m*_ = 30 min (Garnham, 1966), a maturation time of an iRBC of *t*_*m*_ = 24 h (Cox, 1988; De Roode, 2004) and that an infected RBC releases on average *m* = 9 merozoites after bursting (Cox, 1988; De Roode, 2004; Reilly et al., 2007) we find that the observed loss in the proportion of reticulocytes around day 3 p.i. cannot be explained by the increased parasitemia when using the standard parameterization for erythropoiesis (Table 1). This observation is independent of the assumed infectivity of the parasite strain (Supplementary Figure S1) and is also the case if we assume that the infectivity for reticulocytes is substantially higher than for normocytes. This indicates that the reason for the observed decrease in reticulocyte proportion is not mainly due to reticulocytes being parasitized.

It is known that malarial-induced anemia causes erythropoietic suppression, starting during the early stages of infection (Villeval et al., 1990; Sexton et al., 2004; Thawani et al., 2014). By analyzing the expression levels of previously studied genes (Sexton et al., 2004), we found that the fold change in the expression of the genes most strongly associated with erythropoiesis, i.e., α-globin, β-globin major and β-1-globin, can be described by a logistic-loss function given by

(14) $$\begin{array}{l} F(t)=\;\frac{1+e^{-\lambda t_{0}}}{1+e^{-\lambda (t_{0}-t)}} \end{array}$$

where λ defines the loss-rate of gene-expression, i.e., the loss of RBC production and *t*_0_ the time point at which half of the maximal gene expression is reached. We estimate λ = 2.22 d^−1^ (95%-CI [1.31, 3.05]) and *t*_0_ = 3.70 d [3.28, 4.23] (Figure 2A, Table 1). This parameterization is then used to account for malaria-induced modulation of RBC production during the analyses of blood stage infection dynamics in WT and KO infected mice.

### *PbmaLS*_05 (–) merozoites express a reduced infectivity compared to *Pb*ANKA WT

To analyze the infectivity of both parasite strains, we fitted our extended mathematical model (Equations 4–9 with Equation 14) to the experimental data on RBC count, reticulocyte proportion and parasitemia. Additionally accounting for a modulation of RBC production due to infection (i.e., replacing *F*_0_ by *F*_0_*F*(*t*) with λ = 2.22 d^−1^ and *t*_0_ = 3.70 d in Equation 3) improves model predictions, especially regarding the substantial loss in the proportion of reticulocytes starting 3 days p.i. (compare Figure 2B and Supplementary Figure S1A).

By estimating the infectivity for each parasite strain characterized by the rate of infection (β_0_) and the reticulocyte factor (*RF*), our analysis indicates that the WT parasites have a higher preference for infecting reticulocytes than normocytes (Figure 2B and Table 2). During this early infection phase, we estimate a more than 22-fold higher infectivity for reticulocytes than for normocytes i.e., *RF* > 22 (Table 2). In contrast, a similar preference for reticulocytes could not be found explicitly for the KO parasite. Here, a model assuming equal infectivities for reticulocytes and normocytes, i.e., *RF* = 1, performs equally well as a model that assumes a reticulocyte preference (AIC 40.7 vs. AIC 42.7). However, our time courses are too short to clearly identify such a reticulocyte preference for both parasite strains. As a high infection rate β_0_ can be compensated by a small value of *RF* and vice versa, several combinations of β_0_ and *RF* can explain the observed dynamics (Supplementary Figure S2).

**Table 2:** Parameter estimates for parasite infectivity comparing *Pb*ANKA (WT) and *PbmaLS*_05(-) (KO).

**Parameter**	**Unit**	***Pb*ANKA (WT)**	***PbmaLS*_05(-) (KO)**
Infection rate, *β_0_*	×10^−8^ mz^−1^ μl^−1^ h^−1^	(0, 4.84)	7.82 (7.36, 8.31)
Reticulocyte Factor, *RF*		(22.5, ∞)	1
Average Infectivity, β	×10^−7^ mz^−1^ μl^−1^ h^−1^	1.13 (1.08, 1.16)	0.78 (0.74, 0.83)

*Because for PbANKA (WT) only combinations of β_0_ and RF could be determined (structural non-identifiability), only the ranges of the parameters are given. For PbmaLS_05(-), there was no evidence for a reticulocyte preference, i.e., RF = 1. Numbers in brackets represent 95%-confidence intervals of estimates obtained by the profile likelihood method if boundaries could be determined*.

To compare the infectivity of WT and KO parasites, we calculated an average infectivity β based on the estimates of β_0_ and *RF*, which is defined as the infection rate of a single merozoite when placed into the erythropoietic system at the start of infection (see Materials and Methods for a detailed calculation). We find that KO parasites have a reduced average infectivity compared to WT parasites leading to less productive infections (β = 1.13 × 10^−7^ mz^−1^μl^−1^ h^−1^ [1.08, 1.16] for WT vs. β = 0.78 × 10^−7^ mz^−1^μl^−1^ h^−1^ [0.74, 0.83] for KO, numbers in brackets represent 95%-confidence intervals; Table 2). This reduced average infectivity can explain the slower increase in the parasitemia observed for the KO strain (Figure 2B).

If we assume that the infection rate β_0_ does not differ between the two parasite strains, we find a consistently lower reticulocyte factor for the KO compared to the WT (Figure 2C and Supplementary Figure S2). Thus, our analysis indicates that KO parasites might have a particularly impaired ability to productively infect reticulocytes in comparison to the WT during the early erythrocytic stage of infection.

### Parasite infection dynamics under altered erythropoietic conditions

**Figure 3:**
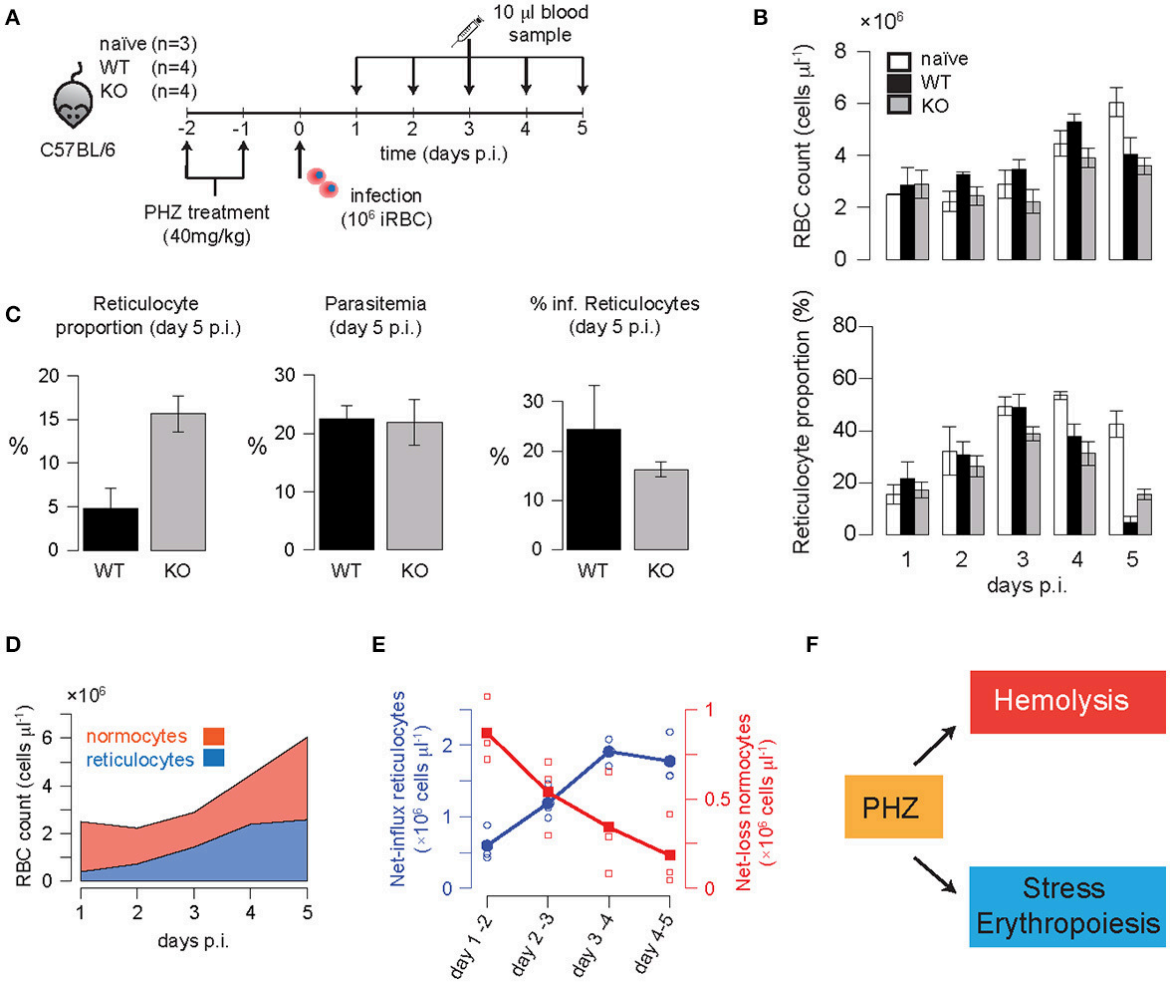
**(A)** Experimental protocol: Mice were pre-treated with two doses of 40 mg/kg PHZ on two consecutive days before infection with 10^6^ iRBC of WT or KO parasites on the following day. Blood samples (10 μl) were taken daily and analyzed. **(B)** Measured concentration of RBC (cells/μl) and reticulocyte proportion for each of the different groups. **(C)** Parasitemia (in % of RBC) above background was detected at day 5 post infection indicating equal levels between WT and KO-infected mice despite a roughly 3-fold higher reticulocyte proportion in KO- compared to WT-infected mice. The percentage of infected reticulocytes was determined as well. **(D)** The measured progression of normocytes and reticulocytes in PHZ-treated but uninfected animals (naïve) indicated an increasing net-influx of reticulocytes (blue line) and a decreasing net-loss of normocytes (red line) up to 5 days post PHZ treatment **(E)**. This corresponds to the assumed effects of PHZ leading to hemolysis and stress-induced erythropoiesis **(F)**.

To elicit possible differences in reticulocyte preferences between the two parasite strains we pre-treated mice with the drug Phenylhydrazine (PHZ) before infecting them with either WT or KO parasites (Figure 3A). PHZ artificially induces anemia in mice causing peroxidation of RBC lipids leading to hemolysis and a change in RBC age distributions (Savill et al., 2009). In uninfected mice that were pre-treated with two doses of 40 mg/kg of PHZ on two consecutive days, we observe a substantial loss in the concentration of red blood cells to roughly ~1/3 of the concentration under homeostatic conditions 2 days after the last treatment with PHZ (2.5 × 10^6^ cells/μl vs. 7.6 × 10^6^ cells/μl, mean values; Figure 3B). There was a corresponding increase in the proportion of reticulocytes to up to 50% of the total RBC count at 5–6 days after the last treatment with PHZ (Figure 3B). Changes in RBC count and reticulocyte proportion of WT or KO infected mice that were pre-treated with PHZ are visible on day 5 p.i. with RBC counts reaching 4.0 ± 0.32 and 3.6 ± 0.15 × 10^6^ cells/μl for WT and KO, respectively, compared to 6.0 ± 0.29 × 10^6^ cells/μl in uninfected animals (Figure 3C). In addition, the proportion of reticulocytes in infected animals is substantially reduced compared to naïve mice; with KO infected mice still having ~3-fold higher levels than WT infected mice [42.6 ± 2.6% (naïve), 4.8 ± 1.2% (WT), 15.6 ± 1.0% (KO); Figures 3B,C]. While parasitemia levels are comparable between both infection groups (22.5 ± 1.2% vs. 21.0 ± 2.0%), the percentage of infected reticulocytes is slightly higher for WT compared to KO (24.3 ± 4.6% vs. 16.3 ± 0.8%; Figure 3C). Given these measurements, the average reticulocyte preference *RP*, calculated by the proportion of infected reticulocytes among reticulocytes divided by the proportion of infected normocytes among normocytes, is determined by *RP*_WT_ = 1.46 and *RP*_KO_ = 0.76, respectively. In accordance with our previous results (Figure 2C), these observations suggest that deletion of *PbmaLS*_*05* has a potential effect on the parasite's ability to productively infect reticulocytes.

### Modeling the effects of PHZ on erythropoiesis and predicting infection dynamics

To determine if the calculated infection characteristics for WT and KO during normal erythropoietic conditions also apply after PHZ treatment, we extended our previous model to account for drug-induced changes to erythropoiesis. The exact mechanisms by which PHZ induces hemolysis and changes in the RBC age distribution have not been determined so far. Several hypotheses including faster aging of RBCs or direct lysis have been suggested and corresponding mathematical models have been proposed (Savill et al., 2009). However, these models fail to fit our experimental data, partly because they are limited to a particular PHZ treatment protocol (Savill et al., 2009). Therefore, we performed a rigorous analysis, testing several different assumptions for the effect of PHZ on erythropoiesis and their ability to explain the observed changes in total RBC count and reticulocyte dynamics in our data (see Materials and Methods and Supplementary Material Text S3 for a detailed description of the different models tested).

**Figure 4:**
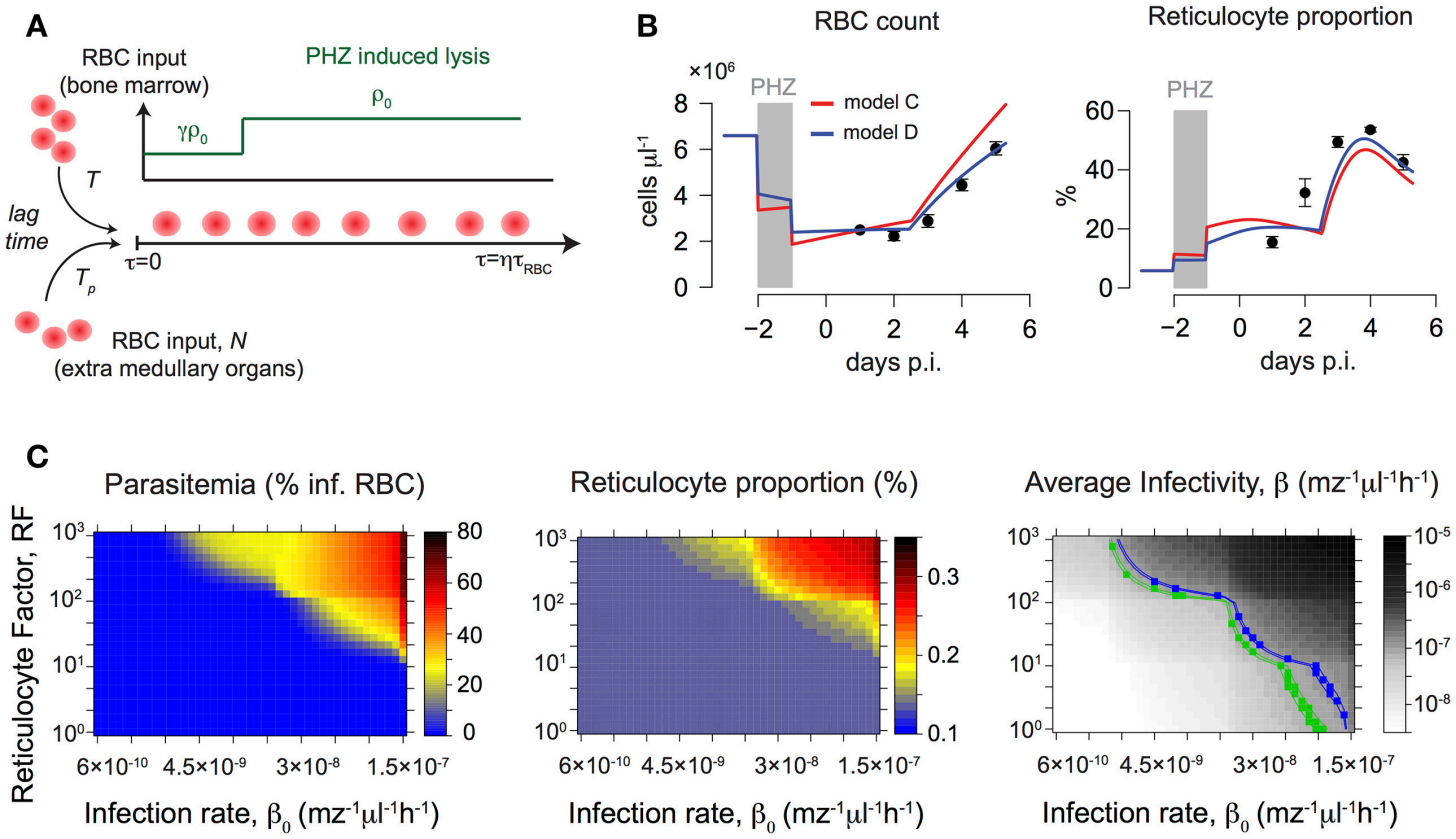
**(A)** Sketch of the mathematical model describing the main effects of PHZ treatment on erythropoiesis. For a detailed description see section Materials and Methods. **(B)** Predictions of the best fit-mathematical model for the dynamics of RBC count and reticulocyte proportion under PHZ-induced changes to erythropoiesis. Gray areas indicate time of PHZ treatment. A model assuming an increased death rate of RBC, 1/τ = 0.125 d^−1^, i.e., due to hemolysis, (blue line) performs better than a model with unchanged RBC lifetimes, 1/τ = 0.025 d^−1^ (red line). **(C)** Predicted parasitemia and reticulocyte proportions after PHZ treatment on day 5 post infection for different combinations of reticulocyte factors, *RF*, and parasite infectivity, β_0_. The right heat-map shows the relevant combinations for WT (blue) and KO (green) leading to the average infectivity as determined during untreated infection (see Figure 2B and Table 1). While for the KO-group relevant parameter combinations lead to matching reticulocyte proportions (~13%) as in the experimental data (compare to Figure 3C), combinations of *RF* and β_0_ for the WT-group predict reticulocyte proportions roughly twice as high as seen in the data.

Our data indicate an increasing influx of reticulocytes, as well as a decreasing net-loss in normocytes after the last PHZ-treatment (Figures 3D,E). Thereby, the increased production of reticulocytes cannot solely be explained by the anemia-induced production from the bone marrow. We found that the best models explaining the effect of PHZ treatment on erythropoiesis assume (i) instantaneous hemolysis with ~35–50% of the RBC being lysed upon PHZ administration, and (ii) stress-induced erythropoiesis with an additional production of reticulocytes from different sources than the bone marrow (Figure 3F). Thereby, this additional production starts around 4.5 days after the last PHZ-treatment has been given (Table 3 and Text S3). In addition, our analysis indicates that PHZ leads to an increased death rate of RBC, reducing the average lifetime of RBC from τ_*RBC*_~40 days to τ_*RBC*_~8 days (see Figure 3E and Table 3). Besides a constant death rate, a linear decreasing death rate, as indicated by our calculation of the observed net-loss in normocytes (Figure 3E), could also be possible as it shows similar explanatory power for the data (Table 3). By incorporating these effects within our model, we are able to provide a modeling framework that describes PHZ-induced changes on erythropoiesis in our experimental system (Figures 4A,B).

**Table 3:** Parameters describing the effect of PHZ treatment on erythropoiesis.

**Model**	**Parameter**	**Unit**	**Value**
With extra-medullary production of RBC	*ρ_0_*	–	0.52 (0.50, 0.54)
	γ	–	0.007 (0, 0.01)
	*T_*p*_*	h	84.5 (83.2, 85.6)
	*N_*p*_*	× 10^4^ cells μl^−1^ h^−1^	7.8 (7.5, 8)
	*r*	–	0.97 (0.92, 0.99)
With extra-medullary production of RBC and constant change in RBC death rate	*ρ_0_*	–	0.38 (0.37, 0.39)
	γ	–	0.006 (0, 0.04)
	*T_*p*_*	h	82.8 (80.2, 84.1)
	*N_*p*_*	×10^4^ cells μl^−1^ h^−1^	7.7 (7.2, 8.1)
	*r*	–	0.92 (0.90, 0.94)
	η	–	4.18 (3.91, 4.46)

*For a detailed explanation of the different models tested to evaluate the different hypotheses for the effect of PHZ see Supplementary Material Text S3. The parameters describe PHZ induced hemolysis (ρ_0_, fraction of RBC lysed; γ, reduction in lysis of reticulocytes) and stress-induced erythropoiesis (T_p_, time delay after PHZ treatment before onset of extra-medullary RBC production; N_p_, rate of RBC influx from extra-medullary sites; r, fraction of N_p_ being reticulocytes). The parameter “η” defines a factor at which the lifespan of RBC produced after treatment is permanently reduced*.

We then simulated the pre-treatment of mice with PHZ and subsequent infection using different assumptions for parasite infectivity, β_0_, and reticulocyte preference, *RF*, and predicted the expected levels of parasitemia and reticulocyte proportion on day 5 post infection (Figure 4C). For the KO strain, relevant parameter combinations as determined previously (Table 2) lead to reticulocyte proportions (~13%) comparable to the ones observed in the experimental data, but result in parasitemia levels of less than 1%. In contrast, combinations of *RF* and β_0_ within the determined ranges for the WT parasite predict reticulocyte proportions that are twice as high as seen in the data (Figure 3C), and parasitemia levels that are only one-tenth of the observed level. However, neglecting previous knowledge and directly estimating *RF* and β_0_ based on the observed parasitemia and reticulocyte proportion under PHZ treatment, both groups expect that nearly all reticulocytes are infected (80–100%), which does not agree with our data (Figure 3C). These findings indicate that there could be disease-induced changes to PHZ treatment effects that cannot be explained by a simple combination of separately determined processes of blood-stage infection kinetics, erythropoiesis and PHZ dynamics.

## Discussion

Parasite replication and invasion of red blood cells during the pathological blood-stage of the *Plasmodium* life cycle is a critical determinant of the severity of disease progression in a malaria infection (Beeson et al., 2016). Determining the precise processes and host factors regulating parasite's infectivity is essential for the identification of appropriate therapeutic targets. Mathematical models have been widely used to understand within-host infection dynamics of the *Plasmodium* parasite through analysis of the complex life cycle and host-parasite interactions in various levels of details (Cromer et al., 2006; Mideo et al., 2008; Li et al., 2011; Kerlin and Gatton, 2013). In this study, we used an age-structured model based on partial differential equations similar to previous approaches (Antia et al., 2008) to specifically determine differences between *Pb*ANKA (WT) and *PbmaLS_05* (-) (KO) parasite strains in terms of age-preferences for RBC, and in particular reticulocytes.

We focused our analysis on the early erythrocytic stage of the parasite, i.e., studying the first 4 days post infection of mice with iRBC. We found that malarial-induced changes to erythropoiesis already play a role at this stage of infection. The observed decrease in reticulocyte proportions could not be explained solely by parasite-induced lysis of RBC (Supplementary Figure S1; Chang et al., 2004), similar to observations for *Plasmodium berghei* at later erythrocytic stages (Cromer et al., 2006). Several factors, including bystander destruction of uninfected RBC during infection (Cromer et al., 2006; Evans et al., 2006; Fonseca et al., 2016) might contribute to the substantial loss in reticulocytes. However, as total RBC counts are rather stable (Figure 2), an age-independent loss of RBC seemed to be insufficient to explain the observed decrease in reticulocyte proportion. Therefore, the mathematical model by Antia et al. (2008) used to describe blood-stage infection dynamics of *Plasmodium* parasites was extended in order to account for altered RBC production dynamics during infection (Sexton et al., 2004; Thawani et al., 2014).

**Figure 5:**
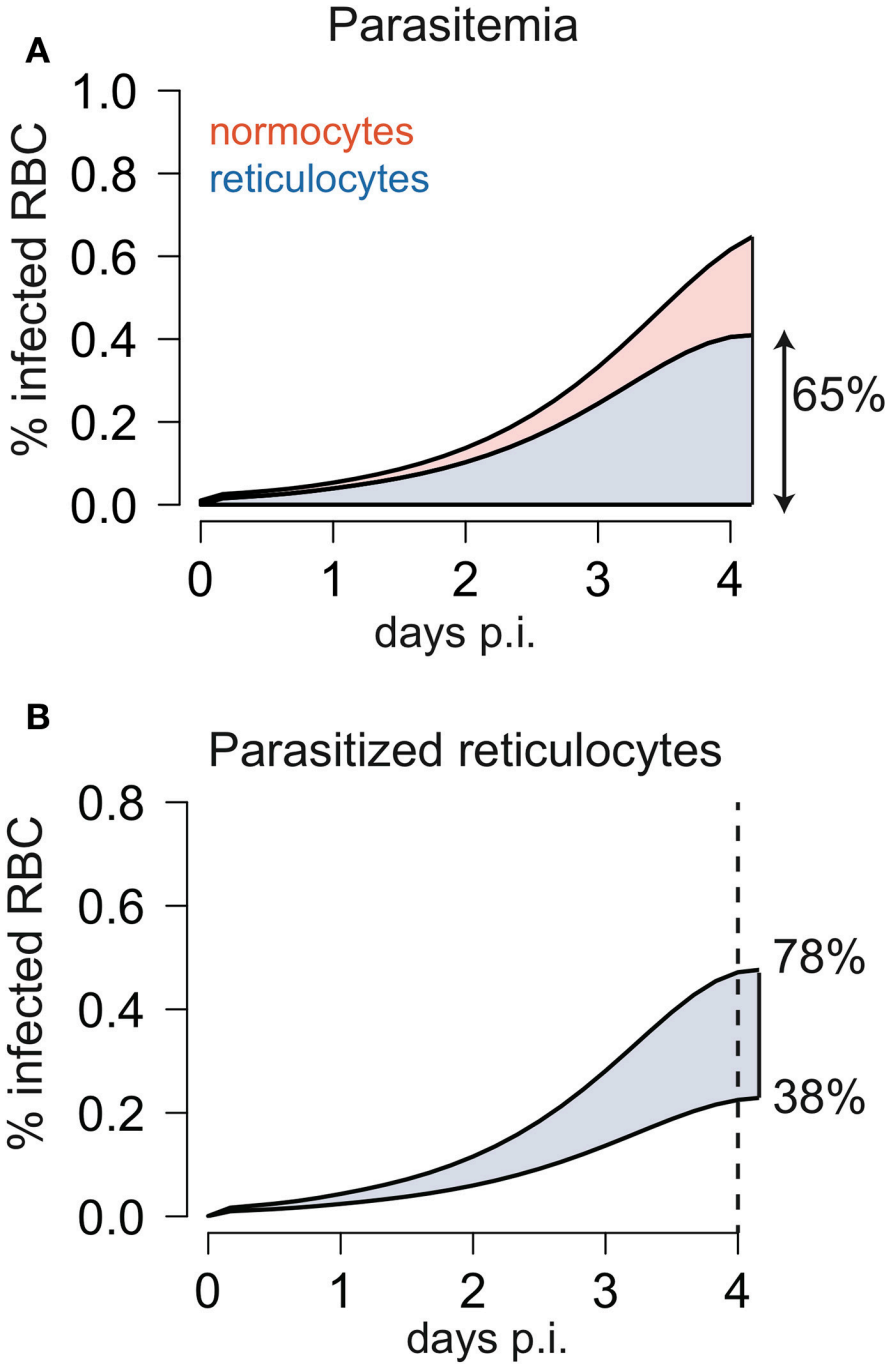
**(A)** Development of infected normocytes and reticulocytes during infection with WT parasites as predicted by the model using a reticulocyte factor of *RF* = 150 as estimated by Cromer et al. (2006). The model predicts that 4 days after infection around 65% of the infected red blood cells are reticulocytes. **(B)** Using the estimated parameter combinations of the infection rate β_0_ and *RF* for *Pb*ANKA (Table 2), the model predicts that 4 days after infection in between 38 and 78% of the infected red blood cells are reticulocytes.

By applying our extended model that disentangles erythropoietic and parasite infection dynamics to the experimental data, we found that *Pb*ANKA prefers to infect reticulocytes. This preference has been observed for various *Plasmodium* strains to different extents (Wilson et al., 1977; Mons et al., 1988; Mons, 1990; Cromer et al., 2006; Antia et al., 2008). We estimate a minimum 22-fold higher preference for reticulocytes compared to normocytes in *Pb*ANKA parasites relying on the early blood-stage of the parasite (Table 2). However, a maximal limit for the *RF* could not be determined (Table 2). As large values of *RF* can be compensated by lower values of the infection rate β_0_, we can only identify combinations of both parameters that would lead to similar levels of parasitemia and reticulocyte proportion (*structural non-identifiability*) (Raue et al., 2009). Thus, even substantially higher values of *RF* could be possible for the WT if the age-independent infection rate β_0_ is accordingly lower (Figure 2C). Cromer et al. (2006) estimated a value of *RF* ~ 150 based on data from later stages of infection with *Plasmodium berghei*, for which a particular reticulocyte preference was found at later times (Singer et al., 1955). With a *RF* ~ 150 as estimated by Cromer et al. (Cromer et al., 2006) our model would predict that infected reticulocytes account for ~65% of the parasitemia at day 4 p.i. (Figure 5). Although this is a slightly larger value than for previous observations in rats infected with *Plasmodium berghei* (Singer et al., 1955), which showed that reticulocytes represent ~50% of the infected RBC on day 4 p.i., such a high reticulocyte factor cannot be excluded based on our analysis.

We also find different combinations of the infection rate β_0_ and the reticulocyte factor *RF* that could explain the observed dynamics for the KO-parasite (Figure 2C). However, we estimate that *Pb*ANKA parasites have a roughly 1.5-fold higher average infectivity than parasites lacking the *PbmaLS*_05 gene (Table 2). Although quite small, this difference is sufficient to explain the observed reduced peripheral parasite load of KO compared to WT infected mice on day 4 p.i. Moreover, the differences between both parasite strains might even be larger than currently estimated. Since WT-infected mice were sacrificed after showing signs of ECM, we were restricted in our analysis to the early exponential growth phase of the parasite in the blood. This could affect the identification of differing parasite infectivities for several reasons: Firstly, parasite levels are still low during this early phase (Figure 1B) and, hence, more prone to measurement noise. Therefore, differences between strains could be masked by the variation in the measurements. Secondly, by using our model to simulate blood-stage infection dynamics assuming various infectivity profiles, we find that differences between infectivity profiles only start to become visible in the measured parasitemia and reticulocyte proportion after 4–5 days p.i. (Supplementary Figure S2). However, comparison of long-term infection dynamics between both strains is hampered as mice infected by *Pb*ANKA WT develop ECM around day 5 p.i.

Based on our analysis, the lower average infectivity for KO compared to *Pb*ANKA WT can be explained by two alternative hypotheses. On the one hand, KO-parasites could have a comparable or larger reticulocyte factor *RF* than the WT, but substantially lower infection rates β_0_ (Figure 2C and Supplementary Figure S2). This would argue for a restriction of the parasite's infectivity to reticulocytes due to deletion of *PbmaLS*_05 (Hopp et al., 2017). In this case, we would expect that such a reticulocyte restriction is particularly visible in mice pre-treated with PHZ, a drug that artificially induces anemia and leads to an increased proportion of reticulocytes. However, we observe a 3-fold higher proportion of reticulocytes in KO- than WT-infected mice 5 days p.i. (Figure 3C). Given comparable levels of parasitemia and total RBC counts, this indicates enhanced reticulocyte survival during infection with the KO-parasite.

Therefore, our analysis rather suggests that deletion of *PbmaLS*_05 impairs the ability of the parasite to productively infect reticulocytes during the early infection phase. The estimated reticulocyte factor *RF* for the WT is around ~1.4 times higher than the one estimated for the KO when assuming similar infection rates (Figure 2C). Furthermore, the calculated reticulocyte preference for KO-infected mice after treatment with PHZ is roughly half the size of the one determined for WT-infected mice. As reticulocytes are usually the preferential targets of parasites (Mons et al., 1988; Mons, 1990), this impaired ability to infect reticulocytes would explain the observed slower increase in parasite burden in mice infected by the KO parasite. In fact, several studies have characterized the need for parasites to infect reticulocytes in order to spread infection. As shown through metabolomic analysis of RBC, reticulocytes possess a higher content of carbon sources and essential nutrients, both of which have been proposed to contribute to the higher reticulocyte preference of WT parasites during the early intra-erythrocytic stages of development (Srivastava et al., 2015). Furthermore, increased expression of CD47 on reticulocytes was shown to prevent phagocytosis and clearance of infected cells (Banerjee et al., 2015), thus allowing unchecked multiplication and infection of new red blood cells. It is therefore plausible that the reduced infectivity of *PbmaLS*_*05* (-) parasites reflected by the parasite's inability to develop within reticulocytes is a major contributing factor to the slower multiplication rates in the blood. Moreover, *PbmaLS_05* (-) infected mice do not develop experimental cerebral malaria but only late stage anemia (Fernandes et al., submitted manuscript), which is in line with previous studies that have proposed a link between severe disease progression and cell preference (McQueen and McKenzie, 2004; Iyer et al., 2007).

In addition to parasite infectivity, we also investigated if the reduced parasitemia in KO infected mice can be explained by impaired merozoite production or altered maturation times for infected RBCs. Assuming similar parasite infectivity for both strains, we do not find evidence for a reduced production of merozoites in KO infection compared to WT (Supplementary Figure S4). However, a roughly 2-fold longer maturation time for iRBC infected by the KO could provide an alternative explanation for the observed differing dynamics (Supplementary Figure S4). This supports the conclusion that deletion of *PbmaLS_05* particularly leads to impaired parasite development and less successful infections in reticulocytes during the initial blood-stage phase.

To fully determine the impact of *PbmaLS_05* deletion on parasite infectivity during the intra-erythrocytic stage and, thus, on disease progression, it remains to be investigated how infection affects erythropoiesis during later phases. Since mice infected with the KO-parasite do not develop ECM, they can be observed for longer time periods. During progression of infection, we observed a substantial increase in the proportion of reticulocytes before mice develop severe anemia and die ~21 days p.i., (Supplementary Figure S5). However, assuming continuous malarial-induced reduction of RBC production, our model is not able to explain the observed dynamics in reticulocytes and parasitemia (Supplementary Figure S5). These observations point toward a recovery of erythropoiesis at later time points, and potentially altered infectivity profiles of the parasite as has been observed for other *Plasmodium* strains. In fact, for the *Plasmodium chabaudi* strain it has been shown that reticulocyte production increases quickly after reaching a minimal production around 9 days after infection with 10^6^ iRBC (Chang and Stevenson, 2004; Chang et al., 2004). In addition, *Plasmodium berghei* has been observed to alter its targeted age range during the progression of infection (Singer et al., 1955; Sexton et al., 2004). Understanding the changes in the erythropoietic processes in the time course of malaria infection remains critical to analyze long-term infection data and to further elucidate the effect of deleting maLS_05 on parasite infectivity and its importance for reticulocyte invasion. This also includes the understanding of the dynamics of infection and reticulocyte development under PHZ treatment. Our analysis revealed that these dynamics are more complex than a simple combination of altered erythropoiesis and infection processes that were parameterized independently.

In summary, our analysis based on a combination of mathematical modeling and experimental data suggests that deletion of *PbmaLS_05* affects productive infection of reticulocytes during the early blood-stage of the parasite's asexual development. Furthermore, our analysis supports previous findings on malarial-induced changes to erythropoiesis that also affect early blood-stage infection dynamics. Given the suggested outcome of *PbmaLS_05* on the productive infection of reticulocytes, we propose that the *PbmaLS_05* (-) mutant parasite strain can serve as a tool to study how the preference of parasites to infect particular RBC influences both disease progression and the development of experimental cerebral malaria. This will ultimately aid in revealing the factors that influence the activation of immune responses and that might enable efficient parasite control.

## Author contributions

Conceived and designed the study: A-KM and FG; Performed the experiments: PF; Developed the mathematical models and analysis methods: NT and FG; Analyzed the experimental data: NT, PF, A-KM, and FG; Wrote the manuscript: NT, PF, A-KM, and FG.

### Conflict of interest statement

The authors declare that the research was conducted in the absence of any commercial or financial relationships that could be construed as a potential conflict of interest.
