# Supplementary material for: Examining the Reticulocyte Preference of Two Plasmodium berghei Strains during Blood-Stage Malaria Infection
Source: Front Microbiol. 2018 Feb 20;9:166. doi: 10.3389/fmicb.2018.00166 (PMC5826286; doi:10.3389/fmicb.2018.00166)
Supplement: Supplementary file 1 [file DataSheet1.pdf]

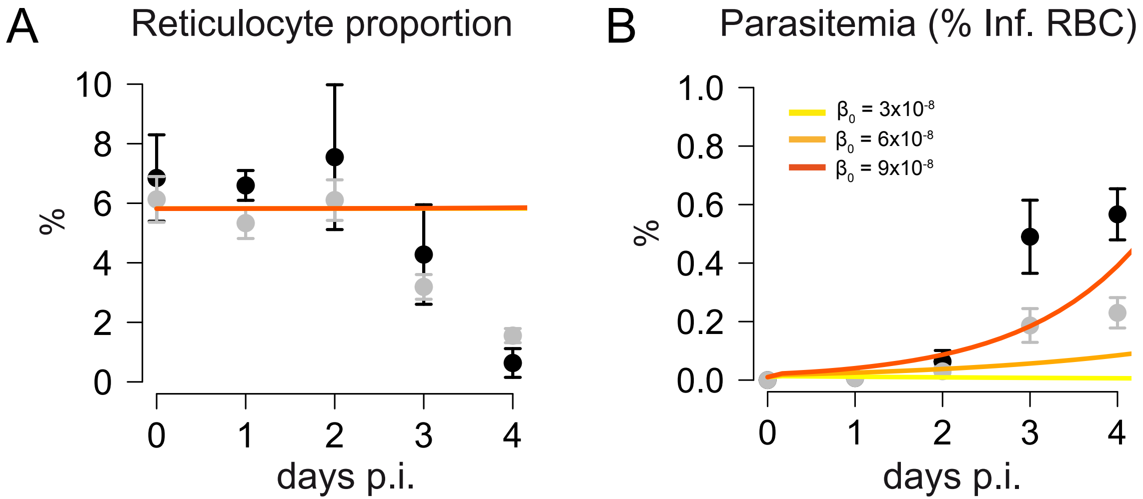

Supplementary Figure S1: Homeostatic erythropoiesis does not explain the observed dynamics in reticulocyte proportion and parasitemia. The observed dynamics of the proportion of reticulocytes (**A**) and parasitemia (**B**) for WT (*black*) and KO (*grey*) parasites are shown. Colored lines indicate the predictions by the mathematical model assuming normal erythropoietic feedback conditions as described by Eqs. (1)-(3) and Eqs. (4)-(8) in the manuscript with different values for the infection rate β_0 (given in mz^{-1} μl^{-1} h^{-1}). Such a model is not able to explain the simultaneous decrease in the reticulocyte proportion and increase in parasitemia around day 3 post infection.
