# Supplementary material for: Examining the Reticulocyte Preference of Two Plasmodium berghei Strains during Blood-Stage Malaria Infection
Source: Front Microbiol. 2018 Feb 20;9:166. doi: 10.3389/fmicb.2018.00166 (PMC5826286; doi:10.3389/fmicb.2018.00166)
Supplement: Supplementary file 2 [file DataSheet2.pdf]

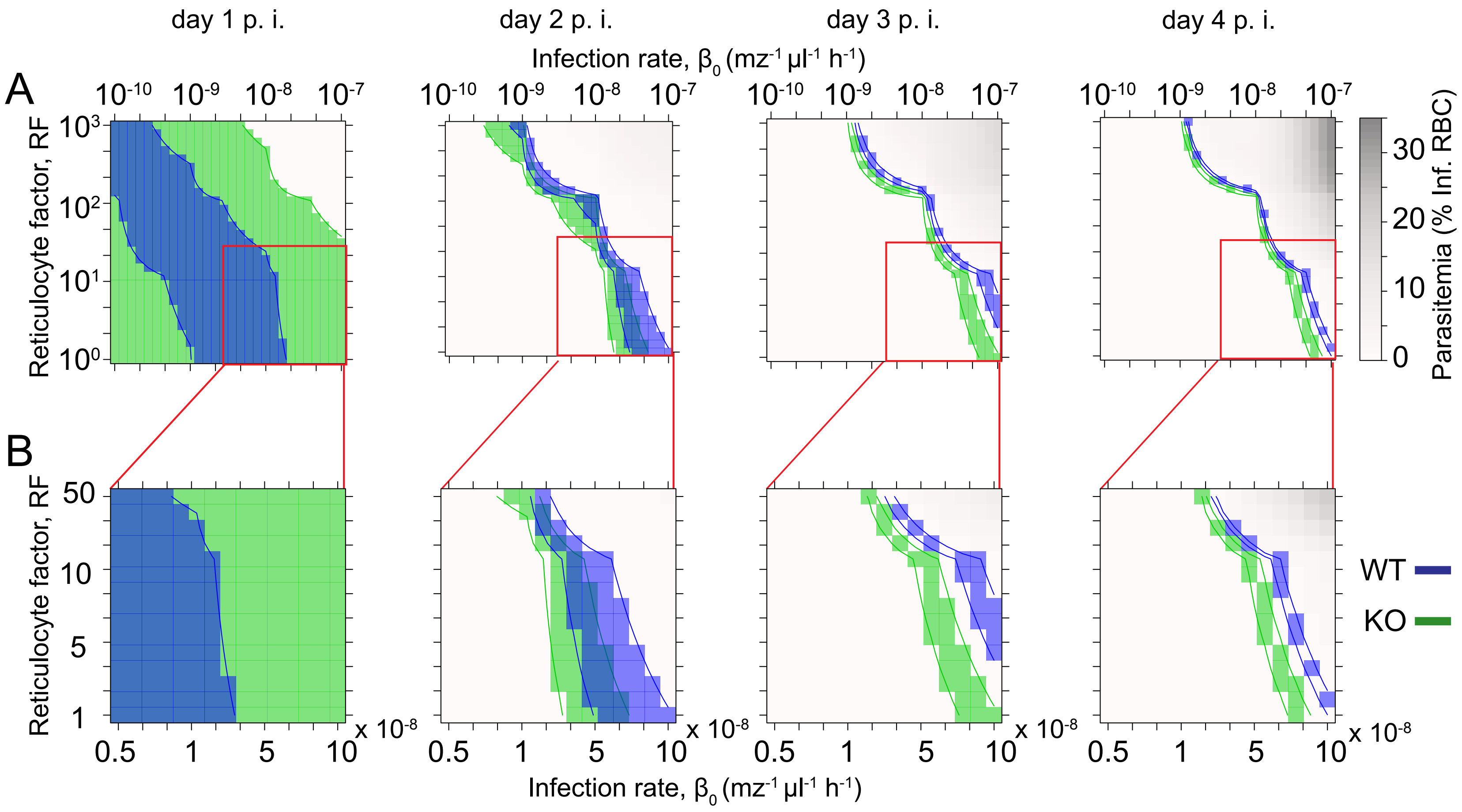

Supplementary Figure S2: Distinguishing between the infectivity of WT and KO parasites during different stages of infection. (**A**) The panels show possible combinations of the infection rate β_0 and reticulocyte factor RF for WT (*blue*) and KO (*green*) parasites explaining the parasitemia level observed. While infectivity characteristics for both parasite strains overlap during early days post infection, later time points allow differing between the two strains. (**B**) Zoom into a particular region of (**A**) to improve visual clarity.
