# Supplementary material for: Examining the Reticulocyte Preference of Two Plasmodium berghei Strains during Blood-Stage Malaria Infection
Source: Front Microbiol. 2018 Feb 20;9:166. doi: 10.3389/fmicb.2018.00166 (PMC5826286; doi:10.3389/fmicb.2018.00166)
Supplement: Supplementary file 3 [file DataSheet3.pdf]

Supplementary Material

Examining the reticulocyte preference of two *Plasmodium berghei* strains during blood-stage malaria infection

Neha Thakre, Priyanka Fernandes, Ann- Kristin Mueller, Frederik Graw

Supplementary Text S3 – Modeling the effect of Phenylhydrazine treatment

Phenylhydrazine (PHZ) is known to induce reticulocytosis, thereby perturbing the age distribution of circulating red blood cells (RBCs). In our experiments, PHZ was used in an attempt to understand how a change in red blood cell age distribution affects the blood-stage infection dynamics of the WT and KO parasite strains.

3.1 Phenylhydrazine (PHZ) and its usage in inducing anemia in mouse models

For many years PHZ has been used for experimental induction of anemia in animal models to study hemolytic anemia or anemia caused by destruction or removal of the RBCs from the bloodstream (1). PHZ causes peroxidation of RBC lipids inducing hemolysis, i.e. the lysis of RBC, which leads to increased levels of extracellular hemoglobin in the blood. Under homeostatic conditions, any hemoglobin produced by hemolysis is reacted upon by certain compounds and is scavenged by macrophages. However, during aggravated hemolysis, the process of scavenging fails to keep up with the increased hemoglobin levels, which in turn leads to a higher level of free heme (2, 3).

The exact molecular mechanisms by which PHZ induces destruction of RBC have not been fully elucidated, and a lot of different processes have been proposed including biochemical modifications, such as ATP decay and increased potassium permeability of cells (4). It has been observed that lysis is usually confined to mature red blood cells (5). Reticulocytes are not lysed although their hemoglobin level is affected (4). Thus, a mathematical model describing the effects of PHZ treatment should consider age-dependent effects of the treatment.

3.2 PHZ induced anemia and the resulting erythropoiesis

Anemia, may it be non-hemolytic (for e.g. induced by bleeding) or hemolytic (for e.g. induced due to a *Plasmodium berghei* infection) leads to enhanced erythropoiesis. In mice, the dominant site for erythropoiesis is the bone marrow. In addition, the spleen also acts as a minor erythropoietic organ. However, in situations that deviate from normal homeostatic conditions, such as anemia due to bleeding or hemolysis, the spleen becomes a major site of erythropoiesis in order to compensate for the loss of RBCs as soon as possible (5, 6). Moreau et al. (3) reported the presence of large numbers of erythroid BFU-E progenitors differentiated from spleen precursors in mice under erythropoietic stress.

As indicated above, treatment by PHZ causes hemolytic anemia and is characterized by the presence of higher levels of free heme in the blood. It has been observed that this anemia results in low hemoglobin levels (which is in positive correlation with the red blood cell concentration, as shown in Fig. 1 from (7)), reticulocytosis (as a result of the feedback) and splenic erythropoiesis (3). It is important to note that Moreau et al. (3) also report a significant lower erythropoietic activity in the bone marrow and a significant higher erythropoietic activity in the spleen during hemolytic anemia as compared to non-hemolytic anemia. This observation points towards considering an additional source compartment for erythropoiesis besides the bone marrow when describing the hemolytic anemia dynamics during PHZ treatment as well as infection due to *Plasmodium berghei*.

3.3 Mathematical models describing PHZ induced treatment effects

There are hardly any studies available around modeling PHZ treatment and the subsequent erythropoietic mechanisms triggered. The work published by Savill et al. (8) studies the effect of PHZ treatment on RBC age-structure. They considered a number of different hypotheses, such as prolonged or immediate effect of PHZ administration and age-specific lysis of RBC to explain RBC dynamics after PHZ-treatment. As these effects did not sufficiently explain our experimental data, we tested several different known hypotheses for the effect of PHZ on erythropoiesis (see Fig. 3.1) and performed a extensive model comparison.

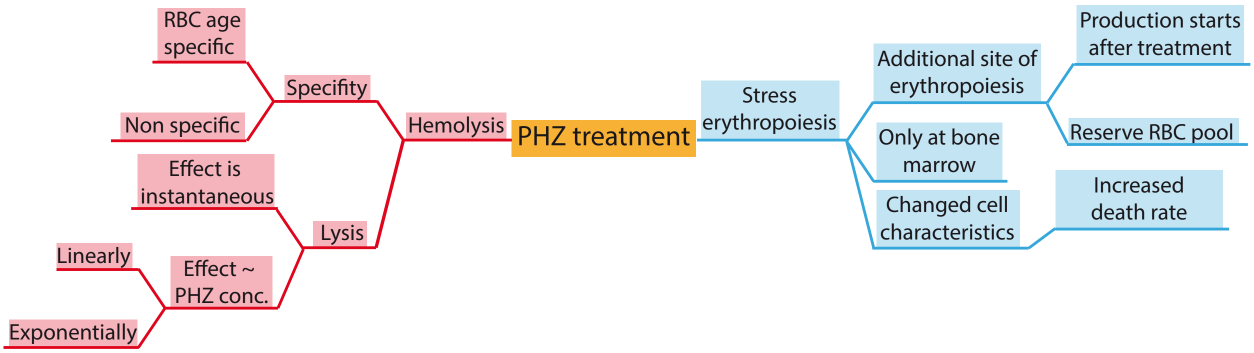

Fig. S3.1 Graph showing the possible effects of PHZ treatment on red blood cells dynamics categorized according to hemolysis (red) and stress-induced erythropoiesis (blue). The different boxes indicate the unknown effects as they are tested within the models.

Here, the effect of PHZ is modeled as an extension to the basic model describing erythropoiesis (see Eq. 1-3 in the main manuscript). The additional model components can be considered to fall into three different categories namely (i) PHZ induced hemolysis, (ii) stress induced erythropoiesis and (iii) altered RBC characteristics (Fig S3.1). Models incorporating different combinations of the various hypotheses describing these effects (see Fig. S3.2) were constructed and tested in their ability to explain the observed dynamics. In the following, five different representative models are explained in detail.

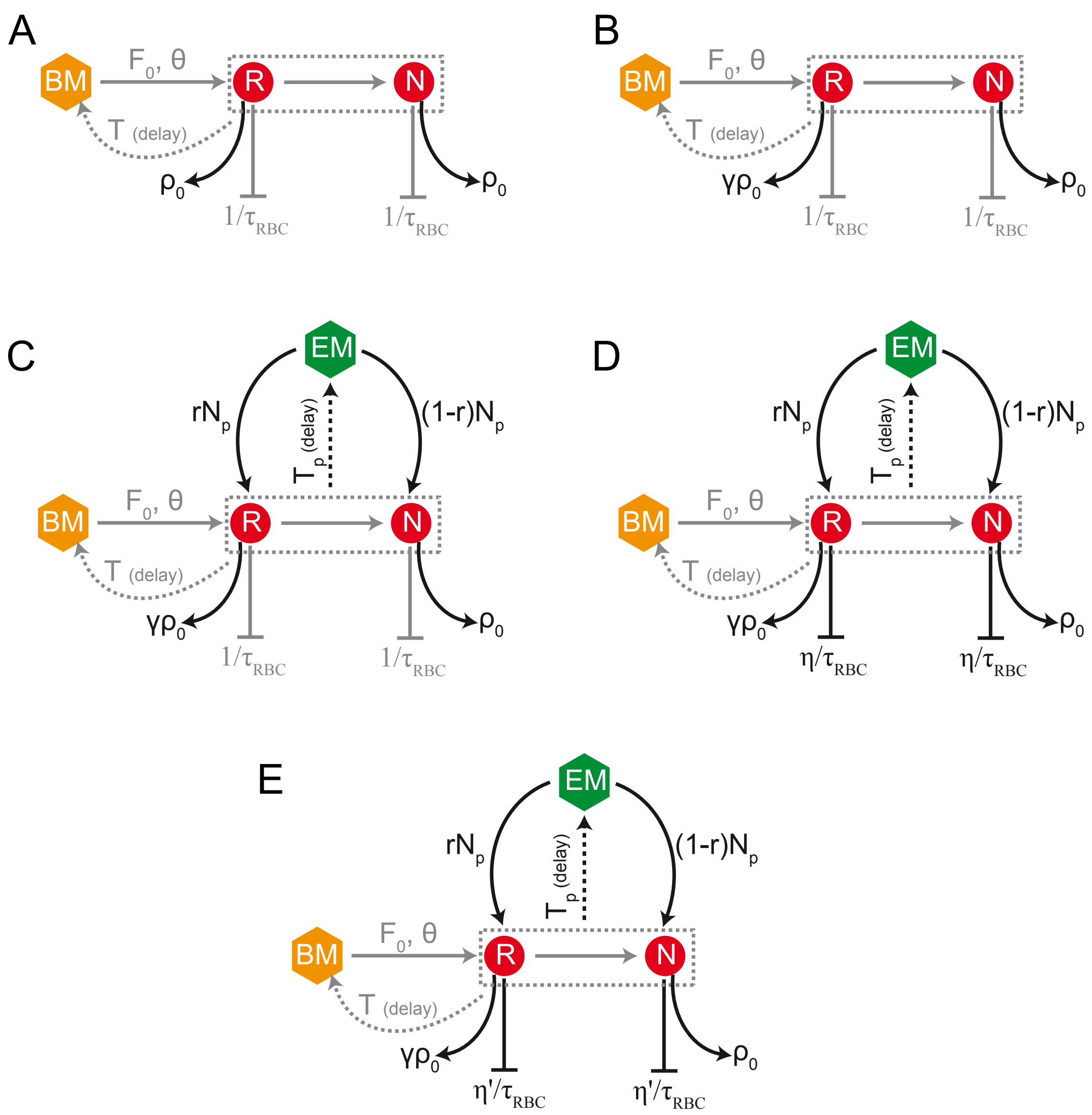

Fig. S3.2 Sketch of the mathematical models including different effects of PHZ treatment on erythropoiesis. Models are shown with increasing complexity from model A-E distinguishing between reticulocytes (R) and normocytes (N) in the blood and the bone marrow (BM) and extramedullary sites (EM). All models and the corresponding parameters are explained in detail below. The standard parameters are shown in Table 1 of the manuscript.

Model A: This is the simplest of all models. The model assumes that the effect of treatment is instantaneous by immediately lysing a fraction, ρ_0**,** of RBCs (irrespective of their age) (Fig S3.2A. Erythropoiesis occurs according to the previously determined conditions (Table 1 in the manuscript), i.e., assuming no stress-induced erythropoiesis. This model fails to explain the excessive increase in reticulocyte proportion and RBC concentration as seen in the data (compare Fig S3.3).

Model B: As in model A, we assume a direct lysis of a fraction, $\rho_{0,}$ of RBC upon administration of treatment, but this time a smaller fraction of reticulocytes γ, as compared to that of normocytes is lysed (8) (Fig S3.2B). As before, we do not assume stress-induced erythropoiesis. Such a model does not improve the fits significantly (compare Fig S3.3).

Model C: In this model, we assume RBC are lysed as in model B. In addition, because of the extreme hemolysis, we assume that stress-induced erythropoiesis contributes to RBC production in addition to RBC production from the bone marrow. Thus, RBCs enter the blood stream from extra-medullary sites modeled with a constant influx of N_p cells μl^{-1} h^{-1}, after a time delay of T_p since the time of treatment. The parameter r with $0 < r < 1$ additionally denotes the proportion of reticulocytes to normocytes that are induced from these extra-medullary sites (Fig. S3.2C). An estimate of r around 1 would mean that RBCs are produced in these organs and an estimate of r around 0 would mean that there is a repository of RBCs in these organs ready to be circulated under stress. Model C significantly improves the fit to our data as it explains the increase in RBC concentration and reticulocyte proportion from day 2 onwards (Fig. S3.3 and Table S3).

Model D: In this model, the assumptions for hemolysis and stress erythropoiesis are as in model C. However, this model additionally assumes that the life span of RBCs that are produced after treatment is reduced permanently by a factor η (Fig. S3.2D). We assume that this change in RBC characteristic is constant over the 5-day period post treatment considered in our experiment. This model extension provides a further improvement to the fit of the experimental data as evaluated by the AIC-value (Fig. S3.3 and Table S3).

Model E: In this model we assume that the effect of PHZ is similar to the assumptions done in model D, however this time the lifetime of RBCs is assumed to recover over time. Meaning, immediately after treatment, RBCs have a reduced lifetime which will increase linearly to the generally assumed lifespan of $\tau_{RBC} = 40$ days over time. The linearly increasing function was pre-determined based on the experimental data, considering the reduction in RBC concentration from one day to the other, the

death rate of RBCs and the reticulocyte maturation time. The parameter η' denoting this increasing life time dependent on the time t is then calculated to $\eta' = -t/30 + 7.4$. Model E only provides a slight improvement compared to model D in explaining the data and leads to an improved AIC-value due to reduced number of free parameters (Fig. S3.3 and Table S3).

Table S3.1: Model performance in explaining the experimental data. The specific parameters that are estimated for each model, as well as the AIC-values indicating model performance are shown. The models with the lowest AIC are generally selected. The latter one is defined as the residual sum of squares divided by the residual degrees of freedom, which is the difference between the number of data points and the number of free parameters.

Model	Parameters to be estimated	AIC (RSS)
**A**	ρ_0	177.7 (10487)
**B**	ρ_0, γ	178.3 (10013)
**C**	$\rho_0, \gamma, T_p, r, N_p$	72.9 (244)
**D**	$\rho_0, \gamma, T_p, r, N_p, \eta$	19.9 (39)
**E**	$\rho_0, \gamma, T_p, r, N_p$	19.4 (41)

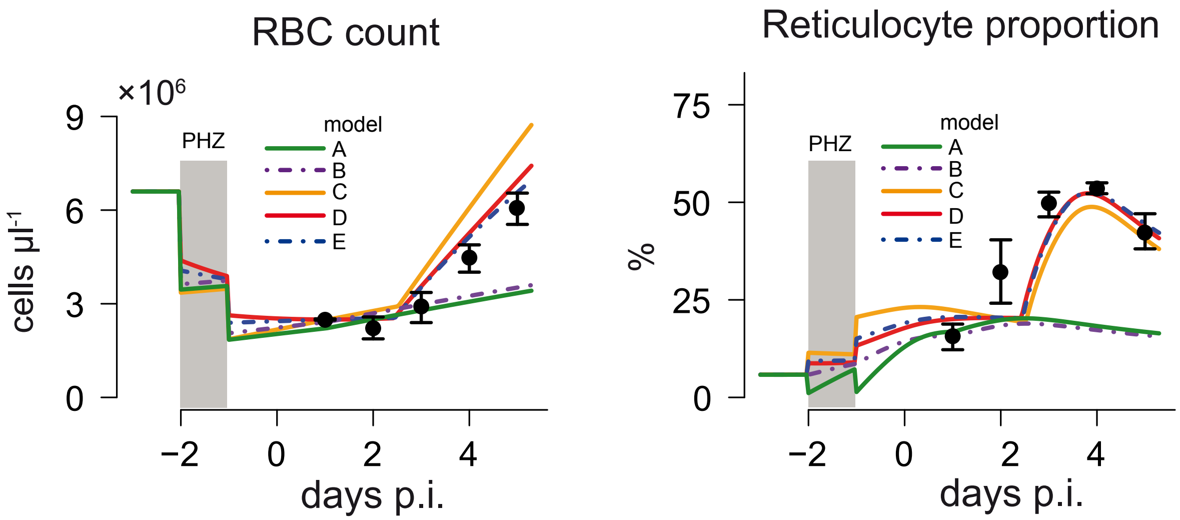

Fig. S3.3: Dynamics of RBC count and corresponding reticulocyte proportion after treatment of C57/BL6 mice with two doses of 40 mg/kg of PHZ at day -2 and -1. The mean and the standard deviation over n=3 mice are shown. Lines indicate the best fits of the models as shown in Figure S3.2. While model A and B are insufficient in simultaneously explaining the dynamics of RBC concentration and reticulocyte proportion after treatment, model C-E including stress-induced erythropoiesis by extramedullary sites significantly improve the fits. In general, model D and E provide the best fits to our experimental model and are therefore considered in the subsequent analyses. See Table S3 for a detailed overview on model performance.

Table S3.2: Parameter estimates for the different models showing best performance in explaining erythropoiesis under PHZ-treatment. For a detailed description of the parameters see the individual model descriptions and Figure S3.2.

Model	Parameter	Unit	Value
**C**	ρ_0	-	0.52 (0.50, 0.54)
	γ	-	0.007 (0, 0.01)
	T_p	h	84.5 (83.2, 85.6)
	N_p	$\times 10^4$ cells μl^{-1} h^{-1}	7.8 (7.5, 8)
	r	-	0.97 (0.92, 0.99)
**D**	ρ_0	-	0.38 (0.37, 0.39)
	γ	-	0.006 (0, 0.04)
	T_p	h	82.8 (80.2, 84.1)
	N_p	$\times 10^4$ cells μl^{-1} h^{-1}	7.7 (7.2, 8.1)
	r	-	0.92 (0.90, 0.94)
	η	-	4.18 (3.91, 4.46)
**E**	ρ_0	-	0.35 (0.37, 0.39)
	γ	-	0.006 (0.002, 0.01)
	T_p	h	83.3 (81.9, 86.1)
	N_p	$\times 10^4$ cells μl^{-1} h^{-1}	7.3 (7.0, 7.9)
	r	-	0.94 (0.89, 0.97)

REFERENCES

1. Berger J. (2007) Phenylhydrazine haematotoxicity. *J. Appl Biomed*; 5:125-30

2. Nielsen MJ, Moller HJ, Moestrup SK. (2010) Hemoglobin and heme scavenger receptors. *Antioxid Redox Signal*.;12(2):261-73.

3. Moreau R, Tshikudi Malu D, Dumais M, Dalko E, Gaudreault V, Romero H, et al. (2012) Alterations in bone and erythropoiesis in hemolytic anemia: comparative study in bled, phenylhydrazine-treated and Plasmodium-infected mice. *PloS One*; 7(9):e46101.

4. Brown KN, Hills LA. (1979) Recent developments in vaccination against malaria: The possible role of isoantigens in protective immunity to malaria. *Bull World Health Organ.*; 57(Suppl):135-8.

5. Spivak JL, Toretti D, Dickerman HW. (1973) Effect of phenylhydrazine-induced hemolytic anemia on nuclear RNA polymerase activity of the mouse spleen. *Blood*.;42(2):257-66.

6. Kim CH. (2010) Homeostatic and pathogenic extramedullary hematopoiesis. *J Blood Med.*;1:13-9.

7. Long PH. (1926) Experimental anemia produced by Phenylhydrazine derivatives. *J Clin Invest.*;2(4):329-42.

8. Savill NJ, Chadwick W, Reece SE. (2009) Quantitative analysis of mechanisms that govern red blood cell age structure and dynamics during anaemia. *PLoS Comput Biol.*; 5(6):e1000416.
