# Supplementary material for: Examining the Reticulocyte Preference of Two Plasmodium berghei Strains during Blood-Stage Malaria Infection
Source: Front Microbiol. 2018 Feb 20;9:166. doi: 10.3389/fmicb.2018.00166 (PMC5826286; doi:10.3389/fmicb.2018.00166)
Supplement: Supplementary file 4 [file DataSheet4.pdf]

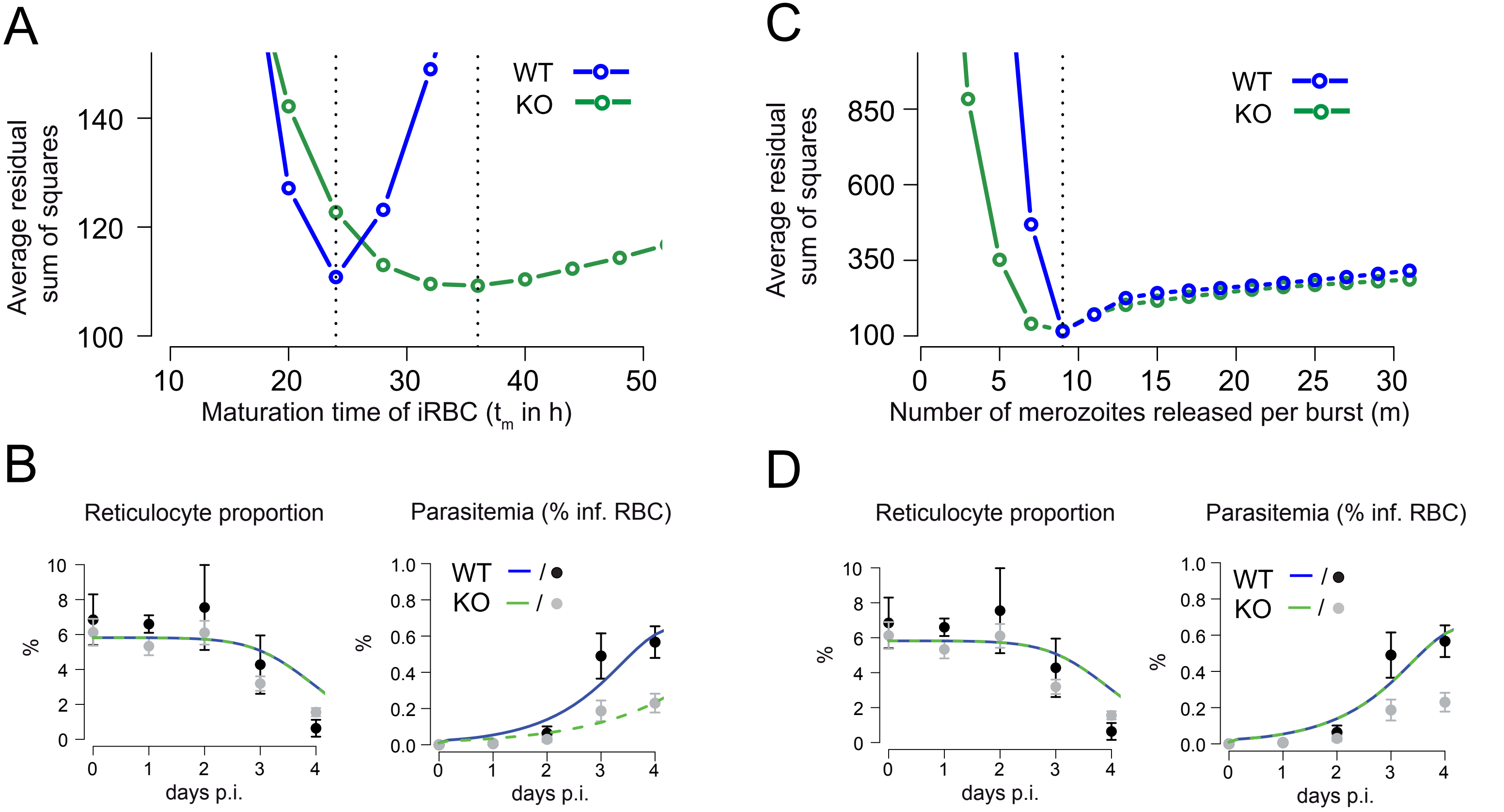

Supplementary Figure S4: Analysis of the experimental data by the mathematical model assuming that the KO differs from the WT strain in the maturation time of infected cells, t_m, or the number of merozoites produced, *m*. In both cases, the infectivity profile for both strains is defined by β_0=4.8×10^{-9} mz^{-1}µl^{-1} h^{-1} and RF= 396.5. (**A**) Profile likelihood analysis for different maturation times of infected cells. Best fits indicate a possible 1.5-fold longer maturation time for cells infected by the KO (*green*, t_m=36h) compared to the WT-parasite (*blue*, t_m=24h). (**B**) Model predictions show good agreement with the observed reticulocyte proportion and parasitemia dynamics. (**C**) & (**D**) Similar analysis for an assumed variation in the number of merozoites produced per infected cell. There is no indication for a difference between WT and KO found within the data. Such a model would lead impaired predictions of the observed dynamics for the KO-strain. In (**B**) and (**D**) black and grey dots indicate the mean and SD at measured time points for the reticulocyte proportion and the parasitemia (**B**).
