# Supplementary material for: Examining the Reticulocyte Preference of Two Plasmodium berghei Strains during Blood-Stage Malaria Infection
Source: Front Microbiol. 2018 Feb 20;9:166. doi: 10.3389/fmicb.2018.00166 (PMC5826286; doi:10.3389/fmicb.2018.00166)
Supplement: Supplementary file 5 [file DataSheet5.pdf]

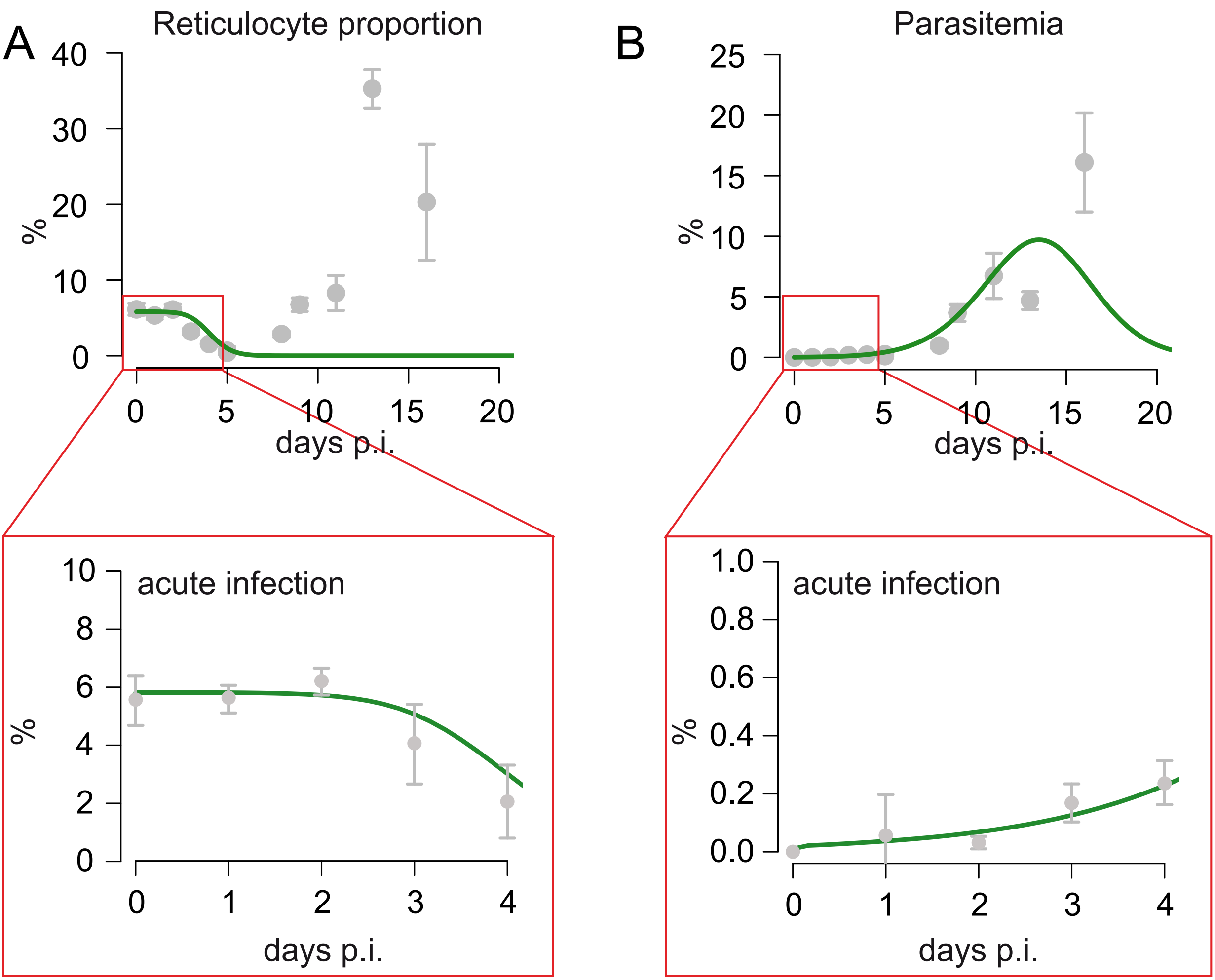

Supplementary Figure S5: Long-term dynamics of the KO-parasite strain followed for 21days post infection when mice develop anemia. The mean and SD at measured time points for the reticulocyte proportion (**A**) and the parasitemia (**B**) is shown in grey. The green line indicates the prediction of our mathematical model for the KO parameterized when fitted to the acute infection dynamics (day 0-4 p.i., lower panels) (see Table 2 in the manuscript). The incapability of the model to explain the observed increase in reticulocyte proportion and parasitemia at later time points indicates a change in the assumed processes regulating erythropoiesis.
